# Defining imagination: a conceptual mapping and provisional model

**DOI:** 10.3389/fpsyg.2026.1888826

**Published:** 2026-09-21

**Authors:** Cassandra Vieten, Julie Eitzen, Chloe Tanega Baxter, Stefanie Holden, Olivia Dance, Corinne Lebrun, Sally Yu, Erik Viirre

**Affiliations:** Arthur C. Clarke Center for Human Imagination, University of California, San Diego, San Diego, CA, United States

**Keywords:** conceptual map, creativity, imagination, mental imagery, visual imagery

## Abstract

**Introduction:**

Imagination is a central feature of human thought, yet it remains poorly defined and inconsistently conceptualized across disciplines, limiting cumulative progress in research. This paper proposes a provisional conceptual model of imagination aimed at organizing existing terminology and clarifying imagination's structure and role in human cognition.

**Methods:**

Using a conceptual review approach, the authors synthesize literature from psychology, neuroscience, philosophy, and related fields, focusing on work that defines, measures, or models imagination. Sources were analyzed to identify recurring components and theoretical perspectives, which were then organized through an iterative process of comparison and integration.

**Results:**

The review identifies 60 dimensions of imagination (in addition to eight forms of applied imagination), organizing these literature-derived terms into three overarching domains: cognitive-narrative, sensory-motor, and social-emotional. Building on these domains, the paper advances a conceptual model in which imagination is defined as the capacity to simulate, reconfigure, and extend beyond reality. The model further proposes that imagination operates across varying levels of awareness and interacts dynamically with attention.

**Discussion:**

This work positions imagination as an integrated, multifaceted process that is deeply embedded in other core mental functions, including memory, planning, and social understanding, rather than as a separate faculty. By offering a framework and clarifying its underlying structure, this conceptual map provides a foundation for future empirical research and highlights the broad significance of imagination in shaping human experience and behavior.

## 1 Introduction

*Imagination will often carry us to worlds that never were. But without it we go nowhere*. ~*Carl Sagan*

Imagination is an essential human capacity. Nearly every human societal advancement springs from a seed of imagination. What we imagine determines our sense of what is possible, which in turn shapes what we do today. Imagination lies at the heart of every building, bridge and road, every innovation and invention, and every social change movement. Empathy and compassion rely on imagining what it might be like to walk in another's shoes. Imagination is also foundational to human flourishing. For example, prospection, or our capacity to imagine and evaluate future possibilities, has been argued to be a cornerstone of psychological functioning and growth (Seligman et al., 2016), and positive mental imagery in particular has been shown to amplify positive emotion and mood more powerfully than verbal thought alone, offering a promising target for interventions that build wellbeing and resilience (Holmes and Mathews, 2005, 2010; Blackwell et al., 2015).

On the other hand, imagination also drives many of our worries, our fears about the future, and our states of anxiety and depression (imagining the worst). Clearly, learning everything we can about imagination, and how it can be applied to building a future that works for all, is a pressing scientific endeavor.

Defining imagination has been a challenge for scholars. *Imago*, the Latin root of imagination, means “to form an image” and derives from the Greek term *phantasia* (fantasy, or imagination). Aristotle in *On the Soul* defined phantasia as the capacity for making mental images, distinguishing it from perception and thinking. When science began to turn its attention to imagination, Galton (1880) asked participants to visualize their breakfast table from that morning and report what they “saw” with as much detail and vividness as they could. Galton called this “mental imagery,” and when he initially tested this questionnaire on scientific colleagues, he was met with baffled responses. There was apparently a lack of ability to visualize or possibly a semantic confusion regarding the phrase “mental imagery.”

“To my astonishment, I found that the great majority of men of science to whom I first applied, protested that mental imagery was unknown to them, and they looked on me as fanciful and fantastic in supposing that the words “mental imagery” really expressed what I believed everybody supposed them to mean…They had a mental deficiency of which they were unaware, and naturally enough supposed that those who were normally endowed, were romancing. (p. 302)”.

When he questioned “general society” though (p. 302), he found people responded with an inherent understanding of visualization. They knew exactly what he was talking about. Overall, Galton found a wide variation in the responses to his breakfast scene questionnaire with some respondents reporting a vivid scene as clear as physically viewing their breakfast table while others reported no ability to visualize their breakfast or anything else.

Experimental work during the early 1900s (Perky, 1910) proposed that imagination was used in a variety of ways and did not have any clear definition, but could be differentiated from memory by its sensory vividness. In 1943, Dexter noted that while the words imaginary (fictitious or fantastic) and imagery (mental pictures) did not seem difficult to define, the term imagination defied consensus definition. Psychoanalyst David Beres defined imagination as a “mental representation of an absent object, an affect, a body function, or an instinctual drive” (1960) which broadened the scientific definition beyond visual or mental imagery.

Current definitions of imagination range from simple to complex. Merriam-Webster (2024) defines it as “the act or power of forming a mental image of something not present to the senses or never before wholly perceived in reality.” Vocabulary.com defines it as “the process of forming images or concepts in the mind, often images of things that are not really there.” Pelaprat and Cole (2011) define it as “a process of image making that resolves “gaps” arising from biological and cultural-historical constraints, and that enables ongoing time-space coordination necessary for thought and action” (p. 397).

Visual imagery remains the most frequently examined form of imagination. It seems clear though, that the definition of imagination refers to much more than visual imagery. The tendency toward a visual bias when it comes to imagination is understandable given the extent to which vision is prioritized in human experience and brain real estate. The dominance of visual forms of imagination in scientific research is further exacerbated by the extensive literature on the visual perceptual system compared to other sensory modalities, and the array of knowledge and tools available to investigate the visual system. Indeed, the word “imagination” itself is based on the word “image.” However, resting the definition of imagination on visual terms is too limited. If imagination is solely defined and investigated as visual imagery, how do we explain the imagination necessary to write a story or think about an alternative ending to a memory recalled from earlier in the week? Thus, a definition of imagination that goes beyond visual is more appropriate for what the term has come to mean.

In recent times, definitions of imagination have become broader. O'Connor and Aardema (2005) frame imagination as a “creative faculty which fills up the possibility space (p. 254),” or the gap between the seen and unseen aspects of human experience. Keltner and Stamkou (2025) define it as “the capacity to construct mental states in the form of beliefs or representations that are not directly perceived through the senses nor present in one's immediate environment” (p. 332). But, important questions about conceptualizing imagination remain. Like other complex brain processes (for example, memory), imagination does not appear to be a siloed operation. It uses other processes to generate an experience, and in turn provides resources to other cognitive and brain processes. For example, working memory likely provides the “mental workspace” to host an imagined event and attention moderates the vividness or intensity of that imagined experience. But reciprocally, imagination likely plays a role in recalling memories. What might imagination's role in perception be? In learning? In empathy or perspective-taking?

A more thorough and precise theoretical synthesis of current conceptualizations of imagination should inform scientific progress, just as emerging findings in cognitive psychology and neuroscience will also inform how imagination should be conceptualized. More precise conceptualization informs hypothesis generation, measurement, methodology, and interpretation of results. In this conceptual review, we discuss what the literature reveals about the roots and facets of imagination, and we propose defining qualities of imagination based on how the field has investigated and defined it thus far. We also synthesize what we have learned and use it as a springboard for a provisional conceptual model of imagination from which to build.

## 2 Materials and methods

The aim of a conceptual review is to develop a deeper understanding of a research topic by synthesizing the current literature from multiple disciplines in order to find connections across the body of research, identify gaps in the research, and develop new hypotheses and/or models. Conceptual reviews are aimed at exploring theoretical frameworks, concepts, and ideas related to a particular topic. It may involve critiquing and synthesizing existing theories, developing new theoretical insights, or proposing conceptual models. Unlike a systematic or scoping review, the goal of a conceptual review is to discuss and integrate different concepts and theories rather than systematically collecting and analyzing empirical evidence. This contrasts with psychometric approaches, such as Zabelina and Condon's (2020) Four-Factor Imagination Scale, which identifies four empirically validated trait dimensions of imagination: frequency, complexity, emotional valence, and directedness, derived through factor analysis rather than literature synthesis.

The purpose of this conceptual review was to determine the primary components of imagination as described in the literature. Our goal was two fold: (1) to identify the component parts of imagination that have been proposed or investigated in both experimental and theoretical work, as well as conceptual models of imagination that have been proposed; and (2) to construct a broad map of the construct and better define imagination as it has been revealed in both theory and empirical study. It should be noted here that this approach has strengths and weaknesses, and should not necessarily result in a conclusive definition or model. A literature review approach to defining a construct risks conflating a construct with its measurement, skewing the definition toward what existing instruments can measure most easily, cultural and publication biases, funding priorities, and more. However, it also provides a starting point that is not purely theoretical, but builds on a common conceptual and empirical foundation, constructing a conceptual map intended to, improve understanding, generate hypotheses, and propose a testable model.

This conceptual review is intended to be argument-driven rather than exhaustive, and is focused on building a case for how imagination should be understood, using literature as evidentiary support rather than as a population to be systematically sampled. The goal is conceptual clarity rather than comprehensiveness. As such, this paper does not claim to have identified every dimension of imagination that exists in the literature, nor to have developed a complete and comprehensive model based on all extant literature. Rather, consistent with the purpose of a conceptual review as described by Hulland (2020), we aim to synthesize existing knowledge in a way that guides readers toward a better understanding of imagination as a construct, identifies gaps in current conceptualizations, and proposes a provisional framework from which future empirical and theoretical work can build.

We focused primarily on material that (1) proposed a measure of imagination with a discussion of conceptualization of the construct and its sub-components, (2) review papers that identified conceptual models or discussed components of imagination, and (3) original research/experimental papers yielding conceptual models or describing components of imagination, and (4) books and book chapters that discussed imagination and its conceptualization, or reviewed or synthesized literature on imagination; and (5) gray literature such as seminar and conference papers, interviews, and science and philosophy blog posts. Material was excluded if it did not define, conceptualize, measure, or examine imagination or one of its components. We included papers from multiple disciplines (psychology, neuroscience, philosophy, etc.) in the form of reviews, editorials, book chapters, and experimental studies.

### 2.1 Data sources and search methods

We utilized PubMed and Google Scholar in our search. The initial search included any articles with title or abstract containing the word “imagination.” All duplicates and articles unrelated to the study of imagination were removed, and remaining articles were screened first by title and then by abstract to assess relevance to the study of imagination. Articles were excluded if they did not define, conceptualize, measure, or examine imagination or one of its components. Following this screening process, 180 full-text articles were included for data extraction.

As we reviewed these articles, additional constructs and terminology related to imagination emerged organically from the literature itself. For example, the term “mental imagery” in one paper led us to trace it across related sources, and similarly for constructs such as episodic future thinking, motor imagery, and mind wandering. This iterative process of following conceptual threads surfaced by the initial review, ensuring that the resulting set of constructs reflected the breadth of the imagination literature as it has actually developed across disciplines. The database search was bounded by electronic indexing availability, with sources dating back to 1969. Key historical sources pre-dating this cutoff, such as Galton (1880), Perky (1910), Dexter (1943), and Beres (1960), were incorporated through citation tracking given their foundational role in conceptualizing imagination.

### 2.2 Data extraction

Five of the authors (CT, JE, SY, OD, CL) performed data extraction. Data extracted from each paper included (1) type of article (review, measure development, original research/experimental, book, book chapter, seminar or conference paper, etc.); (2) measures of imagination used, if any; (3) the type of imagination measured or discussed; (4) components of imagination; and/or (5) theoretical models of imagination proposed. As constructs were extracted, reviewers compiled them into a shared master list. This process was iterative as constructs identified in later articles were compared against those already collected, and similar constructs were grouped together. Importantly, not all constructs encountered in the literature were treated as equivalent components of imagination. During this process, the team actively evaluated whether each construct represented a form or expression of imagination, a product of imagination, or a quality that might modulate imagination, rather than assuming that any construct in the imagination literature was itself a dimension of imagination. Note that we use the term “dimensions” throughout to refer to named aspects or expressions of imagination identified in the literature, rather than in the psychometric sense of latent factors derived from scale analysis.

Supplementary materials Data Sheets 1, 2 document this derivation process, including a working definition, illustrative example, and representative citations supporting each dimension's inclusion, and should be treated as the definitive record of the taxonomy's construction. Table 1 presents a curated, illustrative subset to give the reader an initial sense of how dimensions were defined and categorized into domains and cross-domains.

**Table 1:** Examples of dimensions^*^ of imagination.

Dimension	Definition	Example
Cross-domain (fall into all three domains)		
Dreaming	A subjective experience that occurs when asleep. It can involve all the senses, a range of narrative complexity, and be remembered or forgotten upon awakening	While sleeping, you participate in an elaborate story of living in a castle. You are a knight and have to contend with a neighboring kingdom applying pressure to the Southern border. You balance preparing your army with being in love with the princess
Mental imagery	The experience of sensory information without external stimuli	You imagine yourself walking through the rain in order to reach an underground cavern. On the way, you can see and feel the cold droplets hitting you and the wind pushing against you, your clothes wrapping around your body, even though in reality you are dry and indoors
Mental simulation	The processes, abilities, and/or skills that allow us to create imagined visual images, motor actions, narratives of stories, and generate possible futures	You imagine the discussion you are going to have with your boss later to negotiate a pay raise. You mentally rehearse your pitch, and also include the confidence with which you want to approach your boss. You have a strong but positive tone of voice and a firm handshake. Additionally, you imagine what your boss might say or think in response to your speech so you can best prepare yourself
Mind-wandering	When attention strays from a task at hand to an internal experience, world, or imagining	You sit at your computer working on a paper. While you try to think of ways to strengthen your argument, you remember the trip you are supposed to take to Colorado next week. Suddenly, you are thinking of a documentary you recently watched about a cult in Colorado. Then, you are considering how upbringing influences your personality and vulnerability to manipulation and wonder if you could be convinced to join a cult
Cognitive-narrative		
Conceptual blending	The integration of elements from different domains or subject matters to create a novel concept from other concepts	Describing a slow-moving bureaucratic process as a “soupy delay” blends the sensory qualities of soup (thick, viscous, resistant) with the abstract experience of waiting, producing an emergent sense of murky sluggishness that is present in neither “soup” nor “delay” alone, and that must be actively constructed rather than retrieved from familiar usage (Fauconnier and Turner, 2003; Friedlander, 2024).
Counterfactual thinking	Imagining alternative outcomes or scenarios of a past event	Imagine how your college age son's life would have been different if there had been no COVID outbreak in 2020: how their Zoom graduation could have been different, or their first year of college not having been on Zoom
Creative thinking	The ability to produce novel ideas and unique solutions to problems. To generate a new or remodeled physical or conceptual item or work	An artwork (visual, aural, mixed, literary, etc.) or an improved method for powering vehicles; the ability to think outside the box. Or, on a rainy day, you find yourself without an umbrella so you imagine different alternative ways to stay dry
Cognitive/narrative-social/emotional		
Constructive memory	Memory as a creative process rather than an exact recall of past events	Recalling a doctor's waiting room where you sat for 20 min last week, you're confident there was a stack of magazines on the side table, though in fact the table was empty. Your memory filled in a detail it considered a typical feature of waiting rooms, without your having noticed the substitution or intending to alter anything.
Episodic future thinking	The ability to mentally project oneself into the future in order to construct/experience possible future scenarios, rooted in the retrieval of past memories and combining them with possible novel future events	You consider a future vacation, including where you would go, how you would get there, the length of time you would go for, and where you would stay
Mindreading/ imaginative projection	The act of using one's own mental processes in order to attribute mental states to others and make behavioral predictions about others	You see your friend walk in with a sad expression. You know she had an important job interview earlier in the day, and based on her expression and her general gloomy appearance you can infer that the interview did not go well and she is mentally fixating on what went wrong. You know when she gets sad she likes to rewatch her favorite movie, and you imagine that is what she will be doing later today
Social-emotional		
Emotional imagination	To mentally simulate an emotion or event that brings about emotion	A reader absorbed in a novel may feel genuine sorrow at a character's loss or relief at their narrow escape, demonstrating that imagination can generate authentic emotional responses to fictional circumstances, not only to one's own anticipated experiences.
Imagined interactions	The process of thinking about and mentally rehearsing possible social encounters. This can include verbal exchange as well as any physical interaction or body language	You are nervous about asking your crush out for a date and imagine numerous different ways the social exchange might go. Or a theater audience member watching two characters negotiate a conflict on stage imaginatively tracks and anticipates how their exchange will unfold, engaging in a form of imagined interaction as an observer inhabiting the scene rather than as a participant rehearsing an encounter of their own
Sensory/motor-social/emotional		
Imagined speech	To simulate speaking without use of any physical movements	While preparing for your final project presentation, you envision delivering the speech in front of the class without actually moving your jaw, lips, throat, or tongue
Sensory-motor		
Visual imagery	The act of creating mental images or pictures in one's mind without physical external stimuli	You picture your kitchen exactly as it looks right now-the counters, the cabinets, the window over the sink
Motor imagery	A mental performance of a physical movement	Without moving any muscles in your leg or hip, you imagine yourself kicking a soccer ball in front of you
Touch imagination	Imagining or simulating tactile sensations, including the feeling of touching different textures, surfaces, or objects, without actual physical contact	Imagine yourself holding silk in your left hand and sandpaper in your right. Without actually touching these materials, you can simulate the feeling of touching the two differing textures
Musical imagination	The ability to simulate the experience of music (both novel and existing) without the presence of physical music	After going to a concert, when you go to bed and close your eyes, you still hear the melodies in your head playing repeatedly.
Kinesthetic imagery	Without physically moving, engaging in the sensory experience of performing an action, which can also involve force, effort (regarding muscle movement, balance, and coordination), and spatial sensations	You are a sprinter and you imagine yourself in your starting position, waiting for the whistle. When the imagined race begins, you feel your heart rate increase and your fingers push off the granite track as you fly into action
Sensory/motor-cognitive/narrative		
Embodied imagination/cognition	Feeling, in the body, what you are imagining	You are visiting your friend in the hospital who just broke their foot and femur in a skateboarding accident. Looking at them, you cringe while imagining how it must have felt falling off their board onto the road and the edge of the curb and you feel your own foot and leg emit a pang of pain
Interoceptive imagination	The capacity to mentally simulate, anticipate, or re-create internal bodily sensations, such as breath, heartbeat, tension, warmth, nausea, calm, or hunger, in the absence of the actual physiological stimulus	Imagining the feeling of nausea in response to food poisoning or a noxious substance
Visual imagination	Using visual imagery, this involves creating visual mental representations, but is a broader constructive capacity or activity that uses such representations to create, interpret, transform, or recombine possibilities	You picture a treehouse kitchen with a stove built into a hollowed-out trunk, cabinets woven from branches, and a window looking out onto a floating garden

^*^View the full table of all dimensions, categorized according to the three major domains, in Supplementary material Data Sheet 2. Readers should treat these Supplementary materials, rather than this Table's illustrative subset, as the primary data source.

### 2.3 Data synthesis

Through our review of the literature, all authors worked together to identify and organize dimensions of imagination that have been empirically investigated or theoretically described. The full author team met regularly to discuss extracted dimensions, with meetings focused on two central questions: how do these dimensions relate to one another, and how do they relate to imagination specifically? During this process, the team actively evaluated whether each dimension represented a form or expression of imagination, a product of imagination, or a quality that might modulate imagination, rather than assuming that any dimension appearing in the imagination literature was itself a component of imagination.

Following principles of constant comparative analysis, reviewers met regularly to discuss, refine, and operationally define each dimension. Rather than limiting inclusion based on prevalence in the literature or empirical quality, we prioritized a broad approach to conceptual mapping. Through an iterative, team-based process of discussion and comparison, each dimension was examined, clarified, and refined. We then grouped related dimensions into broader conceptual domains through inductive categorization and consensus building.

Disagreements about the categorization of specific dimensions were resolved through discussion and consensus among the full author team. No dimension was assigned to a domain without agreement, and dimensions whose placement remained genuinely uncertain were flagged for further discussion.

## 3 Results

### 3.1 Search results

Our initial search yielded 337 sources published between the years 1969 and 2023, and after excluding 157 for irrelevance or duplication, the remaining 180 included 46 review articles, 74 original research/experimental papers, six papers testing measures, 23 theory papers, three conference papers, one opinion paper, six books, 12 book chapters, four encyclopedia entries, one review of a book, one response to a critique, two interviews, and one blog entry.

Our components analysis resulted in identifying 60 dimensions of imagination. We created a working definition of each, an example of how it might appear in lived experience, and identified sample citations in the literature. Please see Table 1 for illustrative examples of these dimensions, sorted by a rough approximation of prevalence in the literature. In addition, the full derivation of all dimensions, including working definitions, illustrative examples, and representative citations for each, is available in Supplementary material Data Sheet 1, which we encourage readers to consult as the primary record of how this taxonomy was constructed. Following this, our process of inductive conceptual categorization resulted in organizing these dimensions into three overlapping *domains*: cognitive-narrative, sensory-motor, and social-emotional (see Figure 1).

**Figure 1:**
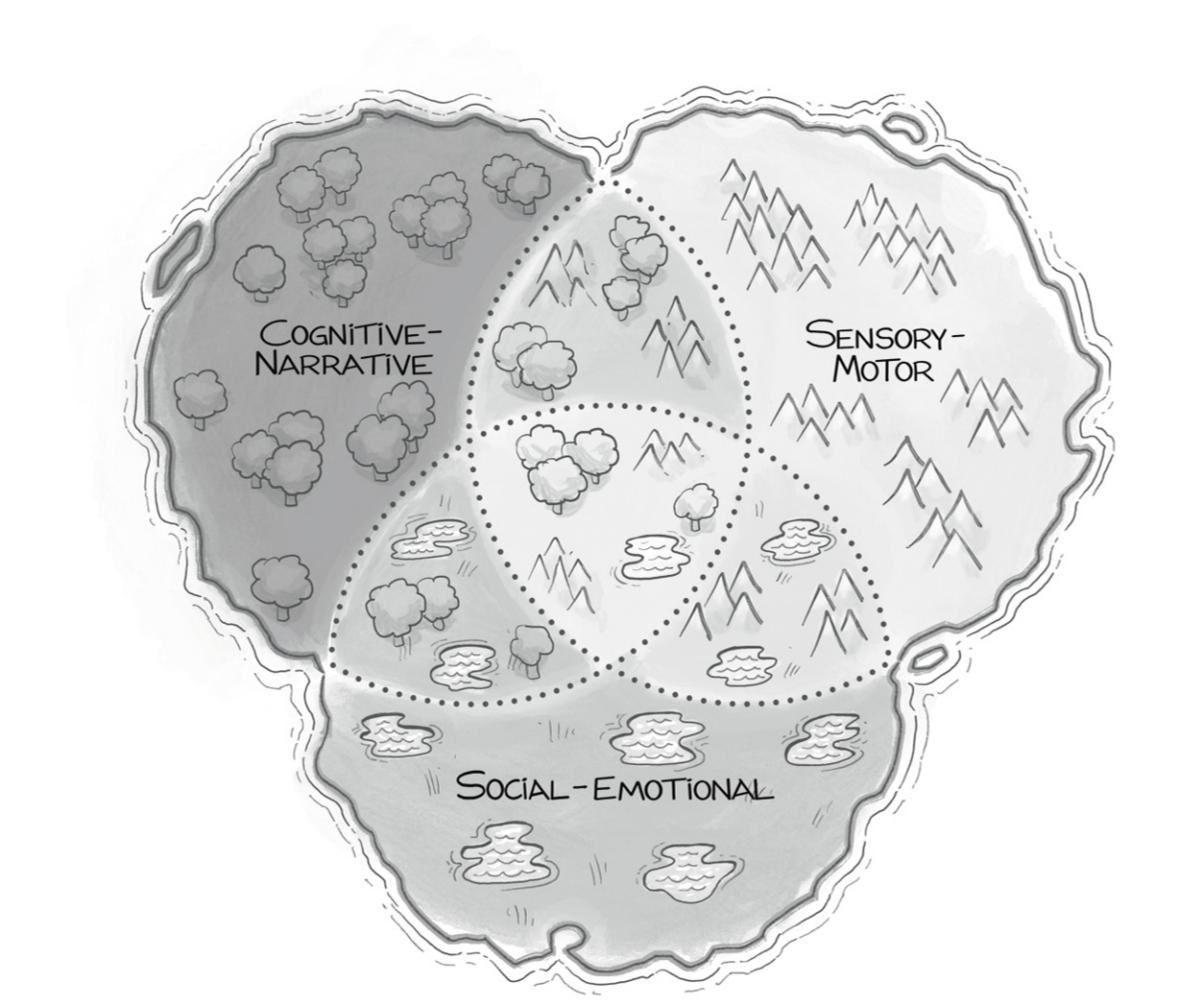
Broad domains of imagination.

### 3.2 Domains and dimensions of imagination

#### 3.2.1 Cognitive-narrative

The Cognitive-Narrative domain of imagination refers to the mental ability to construct, explore, and manipulate complex scenarios that rely on sequential events, including hypothetical, fictional, or future-oriented stories. It plays a critical role in problem-solving, hypothesis generation, storytelling, and the comprehension of stories. This domain includes language-based, storytelling, and logic-centered aspects of imagination. Activities such as counterfactual thinking, purposeful or unintentional confabulation, hypothesis formation, wondering, or conjecturing are included in this domain. The Cognitive-Narrative domain includes the role imagination plays in memory (Schacter et al., 2012), or storytelling about the past. It entails holding representations and/or possibilities in mind in the service of logic and suppositions or hypothetical reasoning (Abraham, 2016; Ball, 2020; Davies and Bicknell, 2016) and problem-solving (Abraham, 2016; McFarland et al., 2017).

Cognitive-narrative imagination also has a temporal, or time-based, component as seen in storyline or plot line development and the cause-and-effect relationship of a narrative which demonstrates how cognitive-narrative imagination encompasses a logic-based pattern. For example, we can imagine a sequence of events unfolding in time (storyline) and predict future events in the sequence with respect to previous events in the storyline.

We can imagine the future and even mentally travel there (Schacter et al., 2017; Suddendorf and Corballis, 2007). Likewise, we can mentally travel to an event that occurred in our own past (Atance and O'Neill, 2001; Baddeley, 2001; Schacter et al., 2017) or mentally represent what it would be like to travel to a time before we were born. All of these examples do not necessarily rely solely upon internal *sensory* representation but also abstract knowledge networks, logic, and the core knowledge of the fundamental rules underlying physics, cause and effect, social cues, and psychology, the frameworks of which may be innate (Spelke and Kinzler, 2007). In this way, we use imagination to wonder and speculate what it may be like to travel to an alternate world in the same way we can use it to theorize in order to generate hypotheses and problem-solve.

Dimensions categorized into Cognitive-Narrative imagination can also lead to social-emotional or sensory-motor experiences, but have been placed in this domain due to their being primarily referred to in the literature as a cognitive-narrative feature. For example, while primarily Cognitive-Narrative, counterfactual thinking, involving the generation of mental representations of alternatives to past or present reality (or pre-factual thinking when referencing the future) (Epstude and Roese, 2010; Schacter et al., 2015), may trigger emotions such as regret, relief, or hope arising as a consequence of the imagined alternative (Coricelli and Rustichini, 2010).

#### 3.2.2 Sensory-motor

Sensory-Motor imagination is defined as having or creating an experience of hearing/auditory, seeing/visual, touching/tactile, tasting/gustatory, smelling/olfactory or other senses such as interoceptive or vestibular, without the need for a sensory stimulus (Belardinelli et al., 2009; Yoo et al., 2003; Croijmans et al., 2019; Djordjevic et al., 2004; Kobayashi et al., 2004; Murphy, 2022). Sensory-Motor imagination also includes imagined movement of self (Yang et al., 2021), other (Jackson and Decety, 2004), or objects (Li et al., 2015), or the sense of overall embodiment in an imagined space, without requiring the presence or sensory perception of actual movement. As opposed to treating imagination as a purely abstract or cognitive process, embodied imagination includes bodily sensations, movement, and interaction with the world in how we generate, experience, and manipulate imagined scenarios.

An example of the sensory part of Sensory-Motor imagination is visual imagery: the internal perception of a visual image without any external visual stimulus (Pearson, 2019; Winlove et al., 2018). We can picture an image, object, or other visual representation while having our eyes closed or open. Auditory or musical imagination is the internal perception of sound or music in the absence of a stimulus (Lima et al., 2016; Martin et al., 2018; Zatorre and Halpern, 2005). In other words, we can figuratively press a play button in our head to “hear” music without the outside, physical presence of music. We can also experience this involuntarily as when a song is stuck on repeat in our heads which is referred to as an “earworm (p. 3)” (Williams, 2015). Motor imagination includes kinesthetic (Callow and Waters, 2005) or motor imagery (Hétu et al., 2013; Kim et al., 2018; Lotze and Halsband, 2006; O'Shea and Moran, 2017), such as a basketball player imagining sinking a three-pointer in their upcoming game, imagining swimming (Beyer et al., 1990), or speech imagery (Nguyen et al., 2017; Tian et al., 2018; Honeycutt et al., 1990). Sensory-motor imagination refers to the experience of auditory, visual, taste, touch, and smell imagery as well as the internal experience of what it would feel like to engage in particular movements. It is integral to planning how to navigate in the real world, concretizing abstract concepts into visualizations, producing musical harmonies, rehearsal of movements, and picturing what the layout of the land may look like when you arrive somewhere.

#### 3.2.3 Social-emotional

Finally, in our model we identify social-emotional imagination as the third major component of imagination. Social-emotional imagination involves simulating emotions, relationships, and social interactions, both real and hypothetical. It includes imagining emotional states in self and others. For example, you might imagine delivering a presentation with being anxious or excited being the emotional component of this scenario. Social-emotional imagination is the affective component of imagination. Social-emotional imagination also includes the emotions and feelings we may experience as the result of social situations and the imagined presence of others (Cocquyt and Palombo, 2023). In the same presentation example, perhaps the main reason for the imagined anxiety or excitement (depending on how you feel about public speaking), is because of the imagined reactions of the audience. Imagining conversations or interactions with others is a common form of social-emotional imagination. Self-talk (Klinger, 1990, 2009) and imagined interactions with others (Honeycutt et al., 2014) are considered to constitute a significant percentage of the time we spend daydreaming. We can also mentally represent the emotional and mental states of others which is seen in social cognition and Theory of Mind (Schaafsma et al., 2015; Schurz et al., 2021). It is critical to understanding how others might be feeling, perspective-taking, and exploring how we might feel about any given imagined incident.

#### 3.2.4 Cross-domain dimensions of imagination

Some dimensions clearly fall into one of the domains and some occupy two of the three domains: they are labeled as (1) cognitive-narrative/social-emotional; (2) sensory-motor/social-emotional; (3) sensory-motor/cognitive-narrative. Still more dimensions were deemed relevant to all three of the broad domains. As applied, these cross-domain dimensions of imagination function in concert. For example, mental storyboarding as employed in creative writing exemplifies the triple-domain overlap: it involves imagined settings, character voices, and bodily actions (sensory-motor), narrative sequencing and atmosphere (cognitive-narrative), and anticipated emotional trajectories for the reader (social-emotional), drawing on all three domains simultaneously (Friedlander, 2024, 2026). Similarly, film previsualization offers a further illustration of this cross-domain integration in an applied setting: a director mentally planning a shot's framing, pacing, depth, and continuity (sensory-motor) in service of narrative sequencing (cognitive-narrative) is simultaneously anticipating the emotional effect of that shot on a viewer (social-emotional). This example illustrates how visuospatial imagination can operate not merely in service of perceptual accuracy, but in support of narrative construction and social-emotional impact (Friedlander, 2026).

Table 1 provides sample dimensions sorted into domains and cross-domains. Supplementary materials Data Sheets 1, 2 provide the full working definitions, illustrative examples, and citations underlying each dimension in this conceptual mapping.

All dimensions categorized into their respective domains and cross-domains can be found in Supplementary material Data Sheet 2. In addition, Table 2 shows all dimensions sorted into their domains and cross-domains.

**Table 2:** Dimensions of imagination sorted into domains and cross-domains.

Category	*n*	Dimensions
Cross-domain	10	Dreaming, mental simulation, mental imagery, creative imagination, mind-wandering, lucid dreaming, daydreaming, aesthetic engagement, constructive imagination, propositional imagination
Cognitive-narrative	9	Creative thinking, process simulation, divergent thinking, counterfactual thinking, conceptual blending, hypothetical reasoning, productive imagination, outcome simulation, goal imagery
Cognitive/narrative–social/emotional	13	Empathy, moral imagination, pretend play, false memory, mindreading/imaginative projection, mental time travel, exploratory talk, episodic future thinking, constructive memory, episodic simulation, reproductive imagination, recreative imagination, functional imagination
Social-emotional	5	Theory of mind, social imagination, imagined interactions, emotional imagination, co-imagination
Sensory/motor–social/emotional	3	Metaphorical imagination, imagined speech, perspective-taking
Sensory-motor	9	Visual imagery, motor cognition/motor imagery, musical imagination, kinesthetic imagery, dynamic imagery, auditory imagination, olfactory imagination, taste imagination, touch imagination
Sensory/motor–cognitive/narrative	11	Visual imagination, mental rotation, spatial imagery/spatial consciousness, verbal imagery/inner speech, embodied imagination, linguistic imagination, internal visual imagery, external visual imagery, experiential imagination, creative visual imagination, interoceptive imagination

Dimensions are organized into three broad domains (Cognitive-Narrative, Sensory-Motor, Social-Emotional), their pairwise overlaps, and a triple-overlap Cross-Domain category. Applied and disciplinary labels (e.g., consumer, geographical, entrepreneurial imagination) are presented separately in Supplementary material Data Sheet 3 rather than integrated here.

Full working definitions, illustrative examples, and representative citations for every dimension listed are available in Supplementary materials Data Sheets 1, 2.

#### 3.2.5 Applied aspects of imagination

In addition to the dimensions organized into our three core domains, our literature search surfaced a further class of eight forms of imagination as applied within specific professional, disciplinary, or commercial contexts, for example, geographical, scientific, mathematic, entrepreneurial, strategic, requisite (safety/engineering), sociological, and consumer imagination. These terms differ in kind from the dimensions cataloged above: rather than naming a cognitive mechanism, phenomenological modality, or type of imagination, each names a field of practice in which the core domains of imagination, cognitive-narrative, sensory-motor, and social-emotional, are recruited and applied. Consumer imagination, for instance, could be described as an applied instance of prospective simulation, embodied imagery, and anticipated affect directed toward product use or acquisition, rather than a distinct cognitive mechanism in its own right. We present these terms separately, with definitions and citations retained, in Supplementary material Data Sheet 3, rather than integrating them into our domain structure. This list is illustrative rather than exhaustive: numerous other disciplinary labels (e.g., historical, legal, architectural, political, cinematic, or literary imagination) would likely qualify on the same grounds had they appeared with comparable frequency in our search.

### 3.3 Defining imagination

After creating a provisional taxonomy of imagination (listing the types of imagination in the literature and categorizing them into broad domains), the next step was to consider how these might integrate into a working definition and theoretical model of imagination, informed by the many ways it has been approached in the scientific and theoretical literature.

Defining a construct requires more than listing and categorizing its component parts: a good definition should tie together the dimensions of a construct. Imagination has so many diverse dimensions that it can be challenging to identify the thread that connects all the types of imagination, or the latent variable that forms the core of these very different imagination experiences. The present work engaged in a process of construct explication, involving synthesizing diverse perspectives to clarify the boundaries, dimensions, and essence of the construct. Existing definitions, theoretical frameworks, and empirical indicators of imagination as described in the literature were systematically reviewed to distill its core components and arrive at a coherent, operational definition. The resulting working definition is as follows:

**Imagination** is the capacity to simulate reality, reconfigure it, and transcend it, generating possibilities beyond the known through visual imagery, multisensory and embodied experiences, and internal narratives. This capacity can operate deliberately or spontaneously, and within or outside conscious awareness. Each component of this definition is addressed in turn.

**Simulate**: the simulation function of imagination produces representations of things found in the world, preserving the relational structure of what is being represented rather than recombining it into new arrangements. In some ways, memory is imagining the past: reconstructive and constructive accounts of memory (Bartlett, 1932; Schacter and Addis, 2007) describe how remembering itself involves reassembling stored fragments into a coherent whole, yet still in service of representing what plausibly happened or will happen, not of generating genuinely novel combinations. When we imagine what we will do later in the day, we are running a model of reality as it is or would straightforwardly unfold, using past experience and current goals to fill in gaps rather than to depart from the known structure of events.

**Reconfigure**: reconfiguration, by contrast, involves recombining known elements into arrangements that have not previously been experienced together, while remaining within the bounds of what those elements' properties and the rules governing them allow. This corresponds to what Boden (1995) terms combinational and exploratory creativity: the generation of novel (if sometimes improbable) combinations of familiar ideas, and the exploration of new possibilities within an existing conceptual space. Most instances of everyday creative imagining, new solutions, fantasy worlds, films, disease cures, plays, technologies, events, websites, festivals, structures, draw on this recombinatory capacity, assembling constituent parts of known reality into new configurations without altering the rules that govern how those parts behave.

**Transcend**: transcendence differs from reconfiguration in kind instead of degree: rather than recombining elements within an existing conceptual space, it involves altering the constraints or rules that define the space itself, generating possibilities that could not have been reached through recombination alone. This corresponds to what Boden (1995) terms transformational creativity. Imagination in this mode can transcend the known and spend time in the possible, or even the impossible, exploring what could not exist under the rules of the world as currently understood.

How does imagination do all of this? This happens through visual imagery, multisensory and embodied experiences, and internal narratives.

**Visual imagery**: our definition makes a point of calling out visual imagery on its own, even though it is a form of multisensory experience, because 1) visual imagery is the most commonly understood and studied form of imagination and, 2) using visual imagery as examples of imagination is very helpful when communicating to large groups about imagination as most people can easily engage in this form of imagination.

**Multisensory and embodied experiences**: but imagination is not limited to visual imagery. Imagination also includes representations of sensory information—the method by which we come to know much of the world around us—that include all of the senses, the five conventional senses plus others like proprioception (the body's ability to sense its own position and movement), interoception (the sense of the internal state of your body or signals from within, like hunger, thirst, heartbeat, breathing, etc.), emotion, mood tone, cold-sweat, goosebumps, and more. This allows for a more fully embodied imagined experience, such as how imagining a piece of music evokes emotion, how imagining a loved one makes you feel warm and fuzzy, how imagining a completely different fantasy world (let's say, where beings interact with the real world via avatars that represent their inner natures) can make you feel wonder and excitement.

**Internal narratives:** finally, imaginative narratives are important to include as one of the primary expressions of imagination because some people seem to have non- or low-sensory imaginations, but have very strong storytelling or abstract, symbolic imaginations. In addition, while one may keep all your ideas to oneself, as a human being, most of us are storytellers. We constantly figure out how to fit the play-by-play of our daily experiences into our own personal narrative (Rovetta, 2024). As a routine part of most people's cognitive functioning, we internally incessantly narrate and embellish our actual or expected experiences: how things may turn out after work when we get home, what might happen if we win the lottery this time, if our significant other has completed the chores we requested they tackle, what our pre-schooler did at school that day, how an upcoming meeting might go, deciding that a coworker looked at us “like that” earlier today because they must dislike us, etc. About these narratives, McAdams (2011) says they are, “a selective reconstruction of the autobiographical past and a narrative anticipation of the imagined future that serves to explain, for the self and others, how the person came to be and where his or her life may be going (p. 99).” Most of these stories involve ourselves, but we also imagine stories that are not necessarily directly focused on ourselves, like movies we create: about whether aliens exist, what happened in ancient Egypt or when the dinosaurs lived, the lives of animals, or what would happen if a non-dominant culture took over.

Imagination plays a role in daydreaming, which may be readily apparent given the internally generated nature of mind-wandering experiences, but it is also a component of how everyday situations and events are perceived. When the information needed to make a satisfactory appraisal of a situation is incomplete (which is often the case), individuals generate hypotheses that help explain circumstances and surroundings in which they find themselves. This process is analogous to visual perception. Much of visual experience involves the brain “filling in” information, based on priors, for regions outside of focal attention, thereby producing the sense of a richly detailed and continuous visual field. At any given moment, a substantial portion of experience is not directly derived from external input, but is instead constructed internally as part of perceptual and interpretive processes.

#### 3.3.1 What imagination is, and is not

This way of thinking about imagination begs the question: how does imagination differ from just plain thinking? Useful conceptualizations in science should not only say what a construct is, but also what it is not. What makes imagining the past different from memory, or theory of mind different from imagination itself?

To address this, we initially attempted to place imagination on a construct map, using conceptual and linguistic analysis to determine how it was similar to and different from adjacent constructs such as memory, theory of mind, belief, and problem-solving. Every attempt to isolate imagination on such a map, or position it along a single axis relative to these constructs, failed. We take this finding seriously rather than treating it as a methodological dead end: we argue it reflects something true about imagination's role in cognition, namely that imagination is not a separate faculty operating alongside memory, theory of mind, or problem-solving. Imagination is the generative, gap-filling mechanism that cognitive processes recruit when they go beyond what is directly known or perceived.

This reframing yields a working criterion, even if it does not yield a hard boundary. Memory is not imagination, but constructive memory recruits imagination when it fills in encoding gaps, reconstructs missing detail, or reassembles fragments into a coherent whole (Bartlett, 1932). This is supported by evidence that remembering and imagining share overlapping functional and anatomical substrates (Schacter and Addis, 2007) and may be instantiations of the same underlying constructive process rather than fundamentally distinct operations (Addis, 2017). Theory of mind is not only imagination, but exercising theory of mind recruits imagination whenever it generates a representation of another person's mental state that is not directly observable, which describes the great majority of everyday mindreading, since access to others' inner lives is always inferential. We include theory of mind and perspective-taking as dimensions within our social-emotional domain not because they are completely comprised of imagination, but because the specific act of constructing an unobserved mental state is an imaginative operation that both depend on. Belief and knowledge are not imagination, but generating a hypothesis that exceeds current evidence recruits imagination in the space between what is known and what is proposed. Problem-solving is not imagination, but generating a candidate solution not already contained in the problem's given information recruits imagination to bridge that gap.

We acknowledge that these boundaries are porous, and in many cases contested in the literature. Scholars disagree about where memory retrieval ends and imaginative reconstruction begins, or where social inference ends and imaginative projection begins. We do not regard this porousness as a weakness of the present account but as an accurate reflection of how imagination actually operates. It does not appear to be a bounded module with clean edges, but as a capacity woven through the fabric of cognition, activated at the specific point where a process must generate rather than retrieve, detect, or deduce.

We propose that even the simulation function of imagination (as well as the reconfigure and transcend functions) differs from memory in that the generation of an image is different from the retrieval of an image. Imagination can rely on retrieval of memories in order to generate images, but *generation* of images may be the key factor that differentiates imagination from other meta-level processes. This invites a further question: is all generation imaginative? We do not think so. What distinguishes imaginative generation is that its output is not bound by its inputs, whereas memory is constrained by the input of past information. Generative imagination constructs content that is not fixed by what is given or retrievable from storage or present in sensory perception. Similarly, deduction is not necessarily imaginative if it only relies on previous information, but deduction can utilize imagination as a tool to go beyond what exists in memory. This is consistent with the broader position that imagination is not a siloed cognitive process but interacts and cooperates with other cognitive processes to produce internal representations of reality.

### 3.4 The dynamics of imagination in awareness

The domains and dimensions described above represent the types of imagination identified in the literature, focusing on variations of imagination and in which domain(s) they operate. However, imagination also varies in another important respect: the degree to which these dimensions of imagination are accessible to conscious awareness. Just as the various dimensions of imagination can be categorized into each of the three domains or their overlaps, they also span a spectrum from fully conscious and deliberate to semi-conscious or entirely outside of awareness. A visual image may arise involuntarily, a counterfactual scenario may begin forming before one is aware of it, a social-emotional imagining may shape behavior without ever reaching the level of conscious reflection.

While our initial approach helped to organize the many types of imagination into categories, it addressed less directly the question of how imagination operates. To that end, a dynamic process model was developed, represented in Figure 2. What was not captured by the domain structure alone was that each dimension of imagination identified can be conscious or non-conscious, intentional or spontaneous. Attention serves as a mediating mechanism in imagination, determining which imaginings are brought into focus and which remain at their periphery or below the threshold of awareness. The model described in this section addresses these dynamics directly, proposing that the relationship between imagination and conscious awareness is dynamic and mediated by attention.

**Figure 2:**
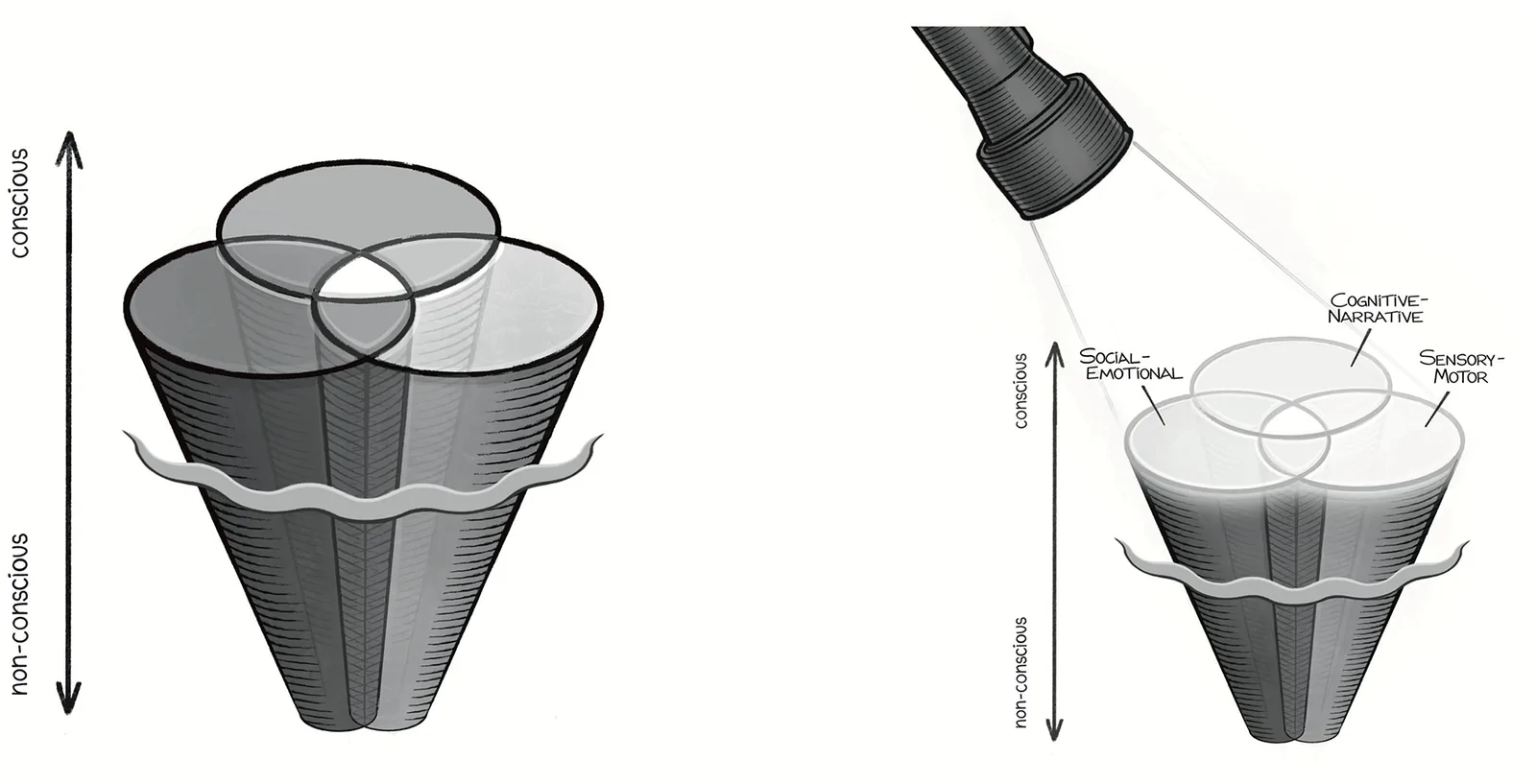
A dynamic model of imagination.

In this visualization of our model (see Figure 2, left panel), a cone sits with its tip pointing downward, like an ice cream cone. It represents a container of consciousness in which imaginings can rise to the surface of awareness. The Venn diagram with three overlapping circles representing the domains of imagination can be conceptualized as the top surface of the cone in this model. Imaginings can rise to the surface and become available to conscious awareness in any one of these categories, or in the overlaps between them. The Venn diagram is the “workspace” where imagination and attention interact (see *Attention and Imagination* below). It is the “place” where imaginings become accessible to us.

#### 3.4.1 Conscious vs. non-conscious

The vertical axis of the cone represents a spectrum of conscious awareness such that the narrowest and lowest point of the cone contains the most inaccessible, non-conscious imaginings. Traveling upwards toward the surface of the cone, imaginings are more likely to be consciously available. When imaginings rise to the surface, they become accessible to conscious awareness. Just below the surface but above the wavy line are semi-conscious and mind-wandering imaginings—not too far away from being conscious, but not immediately in awareness either. These are implicit and not quite within reach, though they may be on their way to being available to conscious awareness.

Below the wavy line lies what we term deep imagining: non-conscious imaginative activity that is the least directly observable and least empirically resolved component of this model, but we surmise is crucial to a complete conceptualization of imagination. We use this term to group three conceptually distinct kinds of material, which we distinguish here rather than treating as equivalent. First, there are non-conscious *processes* that structure imaginative content before it reaches awareness, such as schemas, organizing cognitive structures that shape how incoming and generated information is assembled (Bartlett, 1932). Second, there are non-conscious *contents* that can surface into imagining without deliberate intent, such as dreams and the associative material accessed during them. Third, there are culturally and theoretically derived *constructs*, biases, worldviews, archetypes, and myths, that we include not as claims about a specific cognitive architecture, but as candidate sources of constraint and content that may shape imagining below the threshold of awareness. This may be the most speculative element of the lower layer and a direction for future conceptual and empirical refinement. This layer's theoretical plausibility is supported by Abraham's (2016) quinquepartite classification, which includes an altered-states category comprising imaginative states that stand outside intentional control, including dreams, aesthetic responsiveness, delusions, and meditative states, and links these states to neurocognitive networks distinct from those supporting goal-directed imagining. Whether non-conscious schemas, dream content, and culturally transmitted symbolic material can be shown to influence surface-level imagining in measurable ways remains an open empirical question.

#### 3.4.2 Attention and imagination

Individuals may choose to attend to these imaginings or not. In this model, attention is represented by a flashlight (see Figure 2, right panel). Imaginings enter awareness when they fall within this beam of attention (Posner et al., 1980), or when they “capture” attention due to their salience; that is, when the beam is automatically drawn toward them because they are interesting, loud, or threatening.

Even when the flashlight of attention is directed toward imaginings occurring on the surface of the cone, it may not be possible to illuminate all concurrent activity within imagination at once, given the constraints of attentional capacity. Attention can vary in focus, ranging from narrowly targeted illumination of a single image or domain to a broader beam that encompasses multiple domains simultaneously. For example, composing a musical piece may involve a narrow attentional focus on a specific imagined auditory component, such as a melodic phrase or instrumental timbre. However, as attention broadens, multiple domains of imagination might be engaged in parallel. The sensory-motor domain may support internal simulation of playing or vocalizing musical parts; the cognitive-narrative domain may organize the unfolding structure of the composition as an evolving melodic storyline or “this-and-then-that” sequence; and the social-emotional domain may shape the felt quality or “vibe” of the music, or how it is experienced affectively.

Models such as this serve an important function in conceptual clarification, providing theoretical scaffolding that organizes a complex construct by identifying its key components and the relationships among them. The present model accommodates several aspects of our emerging definition of imagination synthesized from the reviewed literature, including work on memory and future thinking (Addis et al., 2007), research on mental imagery and its influence on conscious perception (Keogh and Pearson, 2018), investigations of the spectrum of imagery ability and its role in processes such as visual working memory (Knight and Milton, 2022; Zeman, 2024), and Langland-Hassan's (2020) model of imagination as the product of a combination of processes rather than a distinct cognitive function.

This model proposes several dynamic features. First, the synthesis of the literature suggests that imagination falls on a spectrum of conscious awareness: it can be conscious, unconscious, or semi-conscious (active in the liminal areas between those two states). Dreams offer a useful illustration. The prevailing theory holds that dreaming involves a form of consciousness, but one largely devoid of self-reflective functions, which may explain why dreams are so often forgotten upon waking (Tononi et al., 2024).

We are cognizant of the risks that philosopher Kind (2021) has identified; allowing for unconscious imagination risks broadening the construct to the point of diminished conceptual utility. However, the present reading of the literature suggests that situating imagination on a spectrum of awareness may more accurately reflect lived experience. Instances of sudden insight-the so-called “Aha!” or “Eureka!” moment-may similarly reflect imaginative processes that had been percolating beneath the surface of awareness before emerging into consciousness. The contention here is that imagination can operate unconsciously, beyond the reach of conscious awareness: what the present model characterizes as beyond the flashlight of attention. The role of attention is therefore central to understanding when and how imaginative experience enters consciousness.

Attention and awareness of imagination also lead directly into whether imagination is considered volitional or intentional, or non-volitional and spontaneous. While the conscious-non-conscious spectrum differs from the volitional/non-volitional spectrum, they are related: imagining on purpose pre-supposes that one is aware of imagining. As noted earlier, experimental findings support the possibility that imaginative processes can precede or operate outside of conscious intention. For example, when individuals with imagery ability were asked to choose between two images to visualize, activity patterns in visual, frontal, and subcortical areas predicted the content of that imagery up to 11 s before any decision was made on what to imagine (Koenig-Robert and Pearson, 2019). This suggests that the neural substrates of imagined content may be established prior to conscious awareness of what is being imagined, rather than arising as a direct product of conscious intention. This volitional/spontaneous distinction has also been operationalized psychometrically: Zabelina and Condon's (2020) FFIS includes directedness as one of four validated trait dimensions of imagination, distinguishing goal-directed from free-floating imaginative states.

Other instances of unintentional imagery include optical illusions, associative imagery, and, again, dreams. Associative imagery is perhaps most strikingly illustrated in the flashback symptomology of PTSD, in which unbidden images and sensations arise outside of voluntary control (Pearson et al., 2015). It has been demonstrated in more subtle perceptual phenomena, such as the experience of a gray-colored tomato appearing faintly red, due to years of learned color association (Hansen et al., 2006). While these examples focus on visual imagery, analogous non-reality based experiences occur across other sensory modalities (Pearson, 2019), as well as in cognitive-narrative and social-emotional forms of imagination. In these domains as well, imaginative processes may operate beneath conscious awareness, similar to non-conscious schemas or biases that shape experience and behavior without deliberate reflection. This remains a promising area for future empirical investigation.

## 4 Discussion

In this conceptual review, we synthesized existing literature to propose a working definition of imagination, an organizing set of component domains and dynamics, and a provisional model of how these operate together. Empirical research, theoretical articles, measures of imagination, books and chapters, and other resources from multiple disciplines were surveyed to create a scaffolding for understanding this complex construct. We present a provisional multidimensional conceptual mapping of imagination in this paper; a subsequent paper will present a further refined synthesis of the many dimensions of imagination and how individual differences in those dimensions might give rise to specific imagination tendencies, styles or profiles.

Synthesizing this literature led to the identification of three broad domains that emerged from the categorization of the imagination dimensions found in the reviewed sources: cognitive-narrative, social-emotional, and sensory-motor. These domains can be visualized as a Venn diagram (see Figure 1). The many types of imagination revealed by the literature review are referred to here as *dimensions* of imagination, sorted into the three broad domains and their intersections according to their definitions, function, and/or phenomenal experience (see Table 1). Some dimensions mapped onto a single domain, while others were more appropriately situated at the intersection of two or all three.

The cognitive-narrative domain is described as a more logic-based or storyline-based domain-the domain in which imagination engages in activities such as counterfactual thinking, hypothetical thinking, or hypothesis generation. The sensory-motor domain is defined as any imagined event that could be perceived by the sensory organs if a stimulus was present, or executed by the musculoskeletal system if one were physically performing a motor movement rather than imagining it (e.g., visual imagery resulting in activity in the visual cortex that, while weaker than that elicited by an external stimulus, mirrors the pattern that would occur if such a stimulus were perceived; Dijkstra et al., 2019). The social-emotional domain is defined as the capacity to incorporate the imagined presence of others, relationships, and feelings, including anticipated thoughts, reactions, and emotional responses, into imagined scenarios. For example, this domain encompasses imagining how others might respond to a new idea, whether and how one might adjust a presentation in anticipation of those responses, and how one feels about others' imagined reactions. It also includes imaginative engagement with the inner lives of others, conjecturing about their potential feelings and experiences.

The proposed model holds relevance beyond taking steps toward definitional clarity. For example, the sensory-motor domain also has direct relevance to psychological wellbeing and intervention. Mental imagery has been shown to function as an emotional amplifier, exerting a stronger causal effect on mood than verbal thought alone (Holmes and Mathews, 2005, 2010), and this property has been leveraged in imagery-based cognitive bias modification interventions, which train individuals to generate positive resolutions to ambiguous scenarios and have demonstrated reductions in anhedonia and depressive symptoms (Blackwell et al., 2015). These findings suggest that the sensory-motor domain of imagination, as defined in the present model, is not merely a descriptive category but a lever with demonstrated applied value for positive psychology and clinical intervention.

Neuroimaging evidence offers partial support for treating cognitive/narrative imagination, social-emotional imagination, and sensory-motor imagination as dissociable, if overlapping, categories: narrative and episodic future-thinking imagination reliably engages a core hippocampal–medial-prefrontal–DMN network that is functionally distinguishable from scene construction proper, with ventromedial PFC engagement scaling specifically with narrative elaboration and temporal extension rather than sensory vividness (Teghil et al., 2025); social-emotional imagination (theory of mind, perspective-taking) recruits a parallel, interdigitated DMN subnetwork anchored in temporoparietal junction that dissociates from the parahippocampal-linked episodic-projection network within the same individuals across repeated scanning (DiNicola et al., 2020); and sensory-motor imagination recruits largely modality-specific circuitry: a pre-motor-parietal network alongside thalamus, putamen, and cerebellum for motor imagery, overlapping substantially with movement execution but not with action observation's cortical-only network (Hardwick et al., 2018). So the three-domain structure has backing in the brain-imaging literature. The categories are separable but not fully modular, since narrative and social imagination still share broader DMN scaffolding and vary in degree rather than kind.

We have proposed that imagination is not its own “module” in the brain but is recruited by many other cognitive processes, a claim that makes brain differentiation nuanced and difficult, though not impossible (Abraham, 2016; Pearson, 2019), and that could easily be the subject of its own review manuscript. Even so, brain science supports the view that imagination is functionally distinct from the processes it draws on. The contextual association network—parahippocampal cortex and retrosplenial complex, responds more strongly to remembered events than to imagined future events, on the logic that remembered events carry richer contextual associations by virtue of having actually happened, while imagined events must be assembled without that scaffolding (Gilmore et al., 2016). At the same time, anterior hippocampus and medial PFC show the opposite pattern, more active for imagined than remembered events, reflecting the added constructive/recombinatorial demand of generating a novel event rather than retrieving one (Addis et al., 2007, 2009). The differentiator, then, is not which regions are involved but which direction their activity moves: posterior contextual regions favor memory, anterior hippocampal/prefrontal regions favor imagination. A parallel dissociation holds for imagination and sensory perception, which draw on the same visual cortex but differ in the direction and strength of the signal: perception is driven by feedforward input that produces strong, decodable stimulus-specific representations, while imagery is generated by top-down feedback that modulates rather than drives activity, producing weaker, less decodable representations in the same early visual areas (Addis et al., 2009; Beaty et al., 2018; Bergmann et al., 2024; Gilmore et al., 2016; Pace et al., 2023).

This distributed, non-modular picture is also consistent with the model's functional versatility: imagination can produce primarily a sensory experience (visual imagery), a propositional experience (problem solving, hypothetical thinking), an emotional experience, or a multimodal experience combining these, interacting with attention, awareness, and a distributed network of brain functions to perform its cognitive roles. This is paralleled at the neural level—attempts to localize imagination to a single discrete brain region have proven unsuccessful, with evidence to date (though limited) instead pointing to a distributed network anchored primarily in the default mode network. Critically, “distributed” does not mean “undifferentiated”: many cognitive processes draw on imagination—memory, problem solving, theory of mind—without being imagination themselves, and imagination in turn is accessed and utilized by these and most other mental functions, working in concert with processes such as working memory and executive function to generate the internal representations of reality (the remembered, the imagined, the possible, the impossible, and their combinations) that give rise to phenomenal experience.

This combination of overlap and distinctness is precisely what the neuroscience evidence above points to, and it is echoed in a different domain by Dijkstra et al. (2019): mental imagery and actual visual perception rely on overlapping neural systems, but differ in function and in their signature patterns of activation. Tateo (2020, 2021) offers a philosophical version of the same duality: rather than treating imagination as an alternative to the cognitive processes associated with knowledge and logic, a diminished form of rationality, or a temporary departure from it, he positions imagination as a complementary and necessary, if not sufficient, form of knowing. In this sense, everything in experience that is not directly perceived through the senses could be understood as a product of imagination, consistent with Stevenson's (2003) characterization of imagination as “the ability to think of anything at all,” suggesting it may function less as a discrete faculty than as a pre-condition for thought itself.

Our three-domain structure is not the first attempt to organize the many senses of imagination, and it is worth situating relative to several influential prior accounts. Abraham's (2016) quinquepartite classification is closest in spirit and aim, proposing five neurocognitively grounded categories: mental imagery-based, intentionality/recollective, novel combinatorial, phenomenology-based, and altered-states imagination. Our cognitive-narrative and sensory-motor domains map reasonably well onto her intentionality-based and imagery-based categories respectively, though our social-emotional domain foregrounds affective and relational content that her classification does not isolate, and her altered-states category corresponds more closely to the awareness/consciousness axis we treat separately in Section 3.4 than to a fourth content domain. Currie and Ravenscroft's (2002) tripartite distinction between creative, sensory, and recreative imagination overlaps partially with our structure. Their sensory imagination aligns with our sensory-motor domain, and their recreative imagination (underpinning perspective-shifting and mindreading) anticipates our social-emotional domain, but their creative category cuts across what we treat as a cross-domain property rather than a standalone domain, and their sustained focus on recreative imagination may have left the creative and sensory categories comparatively underdeveloped, a gap our synthesis could address.

Stevenson's (2003) twelve conceptions, referenced above, serve a different purpose here: rather than proposing functional domains of a single construct, he catalogs distinct philosophical and ordinary-language senses of the word “imagination” itself, spanning epistemic possibility, image-having, non-rational thought, and aesthetic creation; our provisional model may risk oversynthesizing by comparison, but yields a more explicit, potentially measurable operational definition. Finally, Langland-Hassan (2020) account of imagination as an emergent product of coordinated, independent cognitive systems rather than a distinct faculty—the same coordinated-systems view that underlies the distributed picture above—is broadly compatible with our own position; where his account motivates skepticism toward any unified taxonomy, we take the more pragmatic view that a provisional organizing structure remains useful for guiding measurement and hypothesis generation.

The proposed model is intended as a springboard for further development, not a final or complete account of imagination. One valuable next step for this line of research is to go beyond type or variant imagination (the domains and dimensions) to how it works, or its underlying mechanism. The domains presented here group dimensions by type, the kind of experience a dimension typically produces or the context in which it's typically used. But content and mechanism don't always move together: two dimensions placed in different domains because their typical content differs may still share the same underlying cognitive process, while two dimensions sharing a domain because their content is similar may in fact be produced by different mechanisms. The same dimension can also shift in content depending on circumstance, even though the underlying mechanism stays the same. Counterfactual thinking illustrates this: imagining how a dish you intended to order but was sold out would have tasted still begins with cognitive-narrative counterfactual thinking, but can lead to sensory-motor forms of imagination, while imagining how a social misstep might have gone differently remains cognitive-narrative but might lead to social-emotional content. A promising next step, then, might be to build an intermediate layer of categorization, sitting between the broad domains and the full list of dimensions, that groups dimensions by shared mechanism rather than by content, drawing on the mechanism-based work of Abraham (2016), Meyer et al. (2019), and Friedlander et al. (2022). Such a framework would allow the many named types of imagination cataloged here to function as illustrative examples of a smaller set of higher-order processes, representing a promising direction for future theoretical and empirical work.

Models should generate hypotheses that lead to empirical investigations with testable predictions. Ongoing theoretical and experimental work on our proposed dynamic “cone and flashlight” model of imagination could address questions such as: (1) whether non-conscious, pre-conscious, and conscious imagination can be identified in subjective experience, derivative behavior, or neural correlates; (2) whether imagination produced under controlled conditions emerges across the three domains of the proposed model, and whether this represents the most parsimonious schema for categorizing types of imagination; (3) whether attention is volitionally directed toward imaginative content or whether imaginative content directs attention, and under what conditions each occurs; (4) whether imagined content is directional in awareness; whether it “bubbles up” from less conscious levels of processing or, or originates with conscious attention and “sinks down” into non-consciousness.

One limitation of our approach was that our search was not exhaustive, and could have missed concepts important to mapping the construct of imagination. Historical research prior to 1969 was identified only through citation tracking and important historical frameworks could have been missed. Further, our attempt at broad categorization risks an overly simplistic understanding of the complexities. For example, even visual imagery, a dimension within our sensory-motor domain, is itself not unidimensional. Building on Meyer et al.'s (2019) proximal/distal distinction and the ventral/dorsal visual pathway literature, Friedlander et al.'s (2022) Multifactorial Model of Visual Imagery (MMVI) differentiates visual imagery into proximal factors such as episodic/autobiographical imagery, schematic recall, and controlled animation, vs. distal, creativity-linked factors such as inventive combinatorial ability, storyboarding, and conceptual expansion, with only the latter reliably predicting creative achievement. This finer-grained structure illustrates the nuances that likely are at play and should be further unpacked within our broad domains.

Our aim is to advance the state of imagination science and catalyze further theoretical conversation. Deconstructing imagination into its component types and sorting them into domains is an exercise in understanding how imagination operates, what it influences, and how it might be cultivated or optimized. Such concept mapping can refine how the construct is conceptualized, generate testable hypotheses, and inform the development of applications and interventions. Future research should aim to identify the specific neural dynamics that distinguish imagination from related cognitive states, as well as the role imagination plays in various cognitive processes. Given the position advanced here that imagination facilitates diverse cognitive functions, investigating its contribution across a range of such functions would be a logical next step. This could be pursued through studies employing cognitive batteries measuring multiple functions alongside imagination, with modeling techniques such as confirmatory factor analysis or structural equation modeling used to estimate the degree to which imagination contributes to processes such as working memory, attention, language, and visuospatial skills.

Human experience is constituted by streams of thought, sensation, narrative, melody, ideas, and imagery, constantly rising into and receding from awareness. What the senses capture from the external world is imported into a kind of possibility space, where gaps are filled, stories are constructed, and the resulting content populates the continual flow of inner experience. Much of this is made possible by imagination. Understanding what imagination is, how it shapes our perceptions of reality, and how it interacts with other cognitive processes stands to deepen our understanding of this fundamental aspect of being human.
