# Supplementary material for: Defining imagination: a conceptual mapping and provisional model
Source: Front Psychol. 2026 Sep 21;17:1888826. doi: 10.3389/fpsyg.2026.1888826 (PMC13639878; doi:10.3389/fpsyg.2026.1888826)
Supplement: Supplementary file 1 [file Data_Sheet_1.pdf]

Dimension	Definition	Sample Citations	Google Scholar Hits
Interoceptive imagination	Imagining the activity of internal body sensations.	Parra, A., & Rey, A. (2019). The interoception and imagination loop in hypnotic phenomena. Consciousness and Cognition, 73, 102765.<br><br>Wilson-Mendenhall, C. D., Henriques, A., Barsalou, L. W., & Barrett, L. F. (2019). Primary interoceptive cortex activity during simulated experiences of the body. Journal of cognitive neuroscience, 31(2), 221-235.	5
Creative visual imagination	The process of visualizing, transforming, attenuating old mental representations and creating a new one.	Wang, Y., Li, J., Wang, Z., Liang, B., Jiao, B., Zhang, P., … & Liu, M. (2021). Spontaneous activity in primary visual cortex relates to visual creativity. Frontiers in Human Neuroscience, 15.<br><br>Jankowska, D. M., & Karwowski, M. (2015). Measuring creative imagery abilities. Frontiers in psychology, 6, 1591.	146
Functional imagination	Simulate experiences internally without physical actions in order to predict the benefit or consequence in the real world.	Marques, H. G., & Holland, O. (2009). Architectures for functional imagination. Neurocomputing, 72(4-6), 743-759.	168
Touch imagination	Imagining or simulating tactile sensations, including the feeling of touching different textures, surfaces, or objects, without actual physical contact	Ruusunen, N., Hallikainen, H., & Laukkanen, T. (2023). Does imagination compensate for the need for touch in 360-virtual shopping?. International Journal of Information Management, 70, 102622.<br><br>Noheden, K. (2013). The imagination of touch: surrealist tactility in the films of Jan Švankmajer. Journal of Aesthetics & Culture, 5(1), 21111.	194
Co-imagination	Imagining shared among a group. This is commonly encountered in massively multiplayer online role playing games (MMORPG) and table top role playing games such as Dungeons and Dragons.	Aula, I., & Masoodian, M. (2024). Creative Co-Imagination in Transgenerational Comics Workshops. Sociological Research Online, 13607804241259733.<br><br>Bowman, S. L. (2018). Immersion and shared imagination in role-playing games. In Role-playing game studies (pp. 379-394). Routledge.<br><br>Tan, E. S. H., & Visch, V. (2018). Co-imagination of fictional worlds in film viewing. Review of General Psychology, 22(2), 230-244.	247
Experiential imagination	The generation of a mental experience that includes many sensory modalities that mimics being physically present in the mental scene; seeing, hearing, feeling, smelling, etc. parts of the scene/experience.	Clavel Vázquez, M. J., & Clavel-Vázquez, A. (2023). Robustly embodied imagination and the limits of perspective-taking. Philosophical Studies, 180(4), 1395-1420.<br><br>Liefke, K., & Werning, M. (2020, November). Experiential imagination and the inside/outside-distinction. In Jsai International Symposium on Artificial Intelligence (pp. 96-112).<br><br>Kind, A. (Ed.). (2016). The Routledge handbook of philosophy of imagination (Vol. 12). London: Routledge.<br><br>Dokic, J., & Arcangeli, M. (2014). The heterogeneity of experiential imagination. In Open Mind. Open Mind. Frankfurt am Main: Mind Group Springer, Cham.	265
Recreative imagination	Re-creating past perceptual experience in the absence of the stimulus. Rather than "reproducing" a mental photograph, the mind is re-creating an experience in the present	Arcangeli, M. (2022). Chapter: The creative side of recreative imagination; In: The Philosophy of Fiction. Isbn: 9781003139720.<br><br>Langland-Hassan, P. (2020). Explaining Imagination. Oxford: Oxford University Press.<br><br>Kind, A. (Ed.). (2016). The Routledge Handbook of Philosophy of Imagination (1st ed.). Routledge.<br><br>Currie, G., & Ravenscroft, I. (2002). Recreative minds: Imagination in philosophy and psychology. Oup Oxford.	343
Propositional imagination	Accepting that a certain made-up situation or set of circumstances is actually the case. Propositional imagination is usually used in mental exercises such as thought experiments.	Murphy, A. (2022). Imagination in science. Philosophy Compass, 17(6), e12836.<br><br>Nichols, S. (2019). The propositional imagination. In The Routledge Companion to Philosophy of Psychology (pp. 360-369). Routledge.	385
Goal imagery	A mental experience of attaining a personal goal.	Williams, S. E., & Cumming, J. (2016). Athlete imagery ability: A predictor of confidence and anxiety intensity and direction. International Journal of Sport and Exercise Psychology, 14(3), 268-280.<br><br>Chan, C. K., & Cameron, L. D. (2012). Promoting physical activity with goal-oriented mental imagery: a randomized controlled trial. Journal of Behavioral Medicine, 35(3), 347-363.<br><br>Langens, T. A. (2003). Potential costs of goal imagery: The moderating role of fear of failure. Imagination, Cognition and Personality, 23(1), 27-44.<br><br>Schultheiss, O. C., & Brunstein, J. C. (1999). Goal imagery: Bridging the gap between implicit motives and explicit goals. Journal of personality, 67(1), 1-38.	617
External visual imagery	Ability to imagine yourself from a third-person perspective, seeing yourself as the focus of your imagination (rather than a first-person perspective)	Montuori, S., Curcio, G., Sorrentino, P., Belloni, L., Sorrentino, G., Foti, F., & Mandolesi, L. (2018). Functional role of internal and external visual imagery: preliminary evidences from pilates. Neural plasticity, 2018.<br><br>Yu, Q. H., Fu, A. S., Kho, A., Li, J., Sun, X. H., & Chan, C. C. (2016). Imagery perspective among young athletes: Differentiation between external and internal visual imagery. Journal of Sport and Health Science, 5(2), 211-218.<br><br>Callow, N., & Hardy, L. (2004). The relationship between the use of kinaesthetic imagery and different visual imagery perspectives. Journal of sports sciences, 22(2), 167-177.<br><br>Hardy, L., & Callow, N. (1999). Efficacy of external and internal visual imagery perspectives for the enhancement of performance on tasks in which form is important. Journal of Sport and Exercise Psychology, 21(2), 95-112.	732
Taste imagination	The mental simulation or imagining of tastes or flavors.	Xiong, S., Hu, N., & Ma, Y. (2023). The influence of implied motion on expected taste perception of advertised foods: The mental simulation perspective. Food Quality and Preference, 111, 104968.<br><br>Spence, C. (2022). 'Tasting imagination': What role chemosensory mental imagery in multisensory flavour perception?. Multisensory Research, 36(1), 93-109.<br><br>Bensafi, M., Tillmann, B., Poncelet, J., Przybylski, L., & Rouby, C. (2013). Olfactory and gustatory mental imagery: modulation by sensory experience and comparison to auditory mental imagery. Multisensory imagery, 77-91.	775
Olfactory imagination	Imagining a particular odor, or what a particular item/object might smell like.	Flohr, E. L. R., Arshamian, A., Wieser, M. J., Hummel, C., Larsson, M., Mühlberger, A., & Hummel, T. (2014). The fate of the inner nose: odor imagery in patients with olfactory loss. Neuroscience, 268, 118-127.<br><br>Krishna, A., Morrin, M., & Sayin, E. (2014). Smellizing cookies and salivating: A focus on olfactory imagery. Journal of Consumer Research, 41(1), 18-34.<br><br>Bensafi, M., Tillmann, B., Poncelet, J., Przybylski, L., & Rouby, C. (2013). Olfactory and gustatory mental imagery: modulation by sensory experience and comparison to auditory mental imagery. Multisensory imagery, 77-91.<br><br>Djordjevic, J., Zatorre, R. J., Petrides, M., Boyle, J. A., & Jones-Gotman, M. (2005). Functional neuroimaging of odor imagery. Neuroimage, 24(3), 791-801.	813
Internal visual imagery	Visual imagery from the first-person perspective; viewing the imagined scenario as if through your own eyes	Yu, Q. H., Fu, A. S., Kho, A., Li, J., Sun, X. H., & Chan, C. C. (2016). Imagery perspective among young athletes: Differentiation between external and internal visual imagery. Journal of Sport and Health Science, 5(2), 211-218.<br><br>Jiang, D., Edwards, M. G., Mullins, P., & Callow, N. (2015). The neural substrates for the different modalities of movement imagery. Brain and Cognition, 97, 22-31.<br><br>Callow, N., Roberts, R., Hardy, L., Jiang, D., & Edwards, M. G. (2013). Performance improvements from imagery: evidence that internal visual imagery is superior to external visual imagery for slalom performance. Frontiers in human neuroscience, 7, 697.<br><br>Hardy, L., & Callow, N. (1999). Efficacy of external and internal visual imagery perspectives for the enhancement of performance on tasks in which form is important. Journal of Sport and Exercise Psychology, 21(2), 95-112.<br><br>White, A., & Hardy, L. (1995). Use of different imagery perspectives on the learning and performance of different motor skills. British Journal of Psychology, 86(2), 169-180.	1100
Outcome simulation	Contemplating various results and implications of an event, both positive and negative.	Prokopowicz, A., & Byrka, K. (2021). Effectiveness of mental simulations on the early mobilization of patients after cesarean section: a randomized controlled trial. Scientific reports, 11(1), 1-8.<br><br>Li, J., Yang, R., Cui, J., & Guo, Y. (2019). Imagination matters when you shop online: The moderating role of mental simulation between materialism and online impulsive buying. Psychology Research and Behavior Management, 12, 1071.<br><br>Sanna, L. J. (2000). Mental simulation, affect, and personality: A conceptual framework. Current Directions in Psychological Science, 9(5), 168-173.	1360

Dimension	Definition	Sample Citations	Google Scholar Hits
Emotional imagination	To mentally simulate an emotion or event that brings about washes of emotion.	Friedlander, K. J. (2024). The Psychology of Creative Performance and Expertise. Routledge.<br><br>Loev, S., Inchingolo, M., & Velasco, P. F. (2022). What is it (like) to imagine an emotion?. Humanities and Social Sciences Communications, 9(1), 1-9.<br><br>Siclari, F., Baird, B., Perogamvros, L., Bernardi, G., LaRocque, J. J., Riedner, B.,... & Tononi, G. (2017). The neural correlates of dreaming. Nature neuroscience, 20(6), 872-878.<br><br>Hunter, M. (2013). Imagination may be more important than knowledge: The eight types of imagination we use. Review of Contemporary Philosophy, (12), 113-120.<br><br>Moran, R. (1994). The Expression of Feeling in Imagination. The Philosophical Review, 103(1), 75–106.	1450
Linguistic imagination	The capacity to use language generatively, constructing, extending, and transforming meaning beyond the literal through word choice, syntax, narrative, and figuration. It is the ability to deploy language not merely to describe or communicate, but to bring into being possible worlds, perspectives, and meanings that would not otherwise exist	Kearney, R. (2018). Exploring Imagination. Stretching the Limits of Productive Imagination: Studies in Kantianism, Phenomenology and Hermeneutics, 187.<br><br>Dor, D. (2015). The instruction of imagination: Language as a social communication technology. Oxford University Press.<br><br>Miller, J. E. (1970). The linguistic imagination. Elementary English, 47(4), 467-475.	1590
Reproductive imagination	Remembrances; recalling things or events encountered before, like a memory or semantic knowledge. Creating mental images and models from communication with others, reading a book, etc	Boytsova, J. A., Danko, S. G., & Solovjeva, M. L. (2017). States of productive and reproductive imagination reflected in the local synchronization of alpha and theta Eeg frequencies. Human Physiology, 43(3), 353-357.<br><br>Liang, C., Chang, C. C., & Hsu, Y. (2013). Personality and psychological factors predict imagination: Evidence from Taiwan. Learning and Individual Differences, 27, 67-74.<br><br>Liang, C., Chang, C. C., Chang, Y., & Lin, L. J. (2012). The Exploration of Indicators of Imagination. Turkish Online Journal of Educational Technology-Tojet, 11(3), 366-374.	2930
Auditory imagination	To simulate "hearing" a sound without the aid of a physical stimulus. It can be voluntary (call to mind your favorite song and play it in your head) or involuntary (when a song plays over and over in your head without your conscious prompting)	Martin, S., Mikutta, C., Leonard, M. K., Hungate, D., Koelsch, S., Shamma,S., Chang, E. F., del R Millán, J., Knight, R. T., Pasley, B. N. (2018). Neural Encoding of Auditory Features during Music Perception and Imagery. Cerebral Cortex, 28(12), 4222–4233.<br><br>Lima, C. F., Krishnan, C., Scott, S. K. (2016). Roles of Supplementary Motor Areas in Auditory Processing and Auditory Imagery. Trends in Neurosciences, 39(8), 527-542.<br><br>Zatorre, R. J. & Halpern, A. R. (2005). Mental Concerts: Musical Imagery and Auditory Cortex. Neuron, 47(1), 9-12.<br><br>Brodsky, W., Henik, A., Rubinstein, B. S. et al. (2003). Auditory imagery from musical notation in expert musicians. Perception & Psychophysics 65, 602–612.	3000
Embodied imagination	Feeling, in the body, what you are imagining.	Clavel Vázquez, M. J., & Clavel-Vázquez, A. (2023). Robustly embodied imagination and the limits of perspective-taking. Philosophical Studies, 180(4), 1395-1420.<br><br>Langland-Hassan, P. (2020). Explaining imagination. Oxford University Press.	3340
Imagined interactions	The process of thinking about and mentally rehearsing possible social encounters. This can include verbal exchange as well as any physical interaction or body language.	Friedlander, K. J. (2024). The Psychology of Creative Performance and Expertise. Routledge.<br><br>Honeycutt, J. M. (2019). Imagined Interactions and Inner Speech. Imagination, Cognition and Personality, 027623661986916.<br><br>Honeycutt, J. M., & Hatcher, L. C. (2015). Imagined Interactions. The International Encyclopedia of Interpersonal Communication, 1–10.<br><br>Vezzali, L., Capozza, D., Giovannini, D., & Stathi, S. (2011). Improving implicit and explicit intergroup attitudes using imagined contact: An experimental intervention with elementary school children. Group Processes & Intergroup Relations, 15(2), 203–212.<br><br>Honeycutt, J. M. (1990). Imagined Interactions, Imagery, and Mindfulness/Mindlessness. Mental Imagery, 121–128.	3660
Dynamic imagery	Mentally envisioning and perceiving of an action without physically engaging in output	MacIntyre, T. E., Moran, A. P., Collet, C., & Guillot, A. (2013). An emerging paradigm: a strength-based approach to exploring mental imagery. Frontiers in Human Neuroscience.<br><br>MacIntyre, T., and Moran, A. (2010). "Meta-imagery processes among elite sports performers," in The Neurophysiological Foundations of Mental and Motor Imagery, eds A. Guillot and C. Collet (New York, Ny: Oxford University Press), 227–244.	3760
Imagined speech	To simulate going through the motions of speaking without use of any physical movements.	Proix, T., Delgado Saa, J., Christen, A. et al. (2022). Imagined speech can be decoded from low- and cross-frequency intracranial Eeg features. Nature Communications, 13(48).<br><br>Lee, S. H., Lee, M., Lee, S. W. (2020). Eeg Representations of Spatial and Temporal Features in Imagined Speech and Overt Speech. In: Palaiahnakote, S., Sanniti di Baja, G., Wang, L., Yan, W. (eds) Pattern Recognition. Acpr 2019. Lecture Notes in Computer Science(), vol 12047. Springer, Cham.<br><br>Tian, X., Ding, N., Teng, X., Bai, F., Poeppel, D. (2018). Imagined speech influences perceived loudness of sound. Nature Human Behaviour, 2, 225-234.<br><br>Nguyen, C. H., K Karavas, G., Artemiadis, P. (2017). Inferring imagined speech using Eeg signals: a new approach using Riemannian manifold features. Journal of Neural Engineering, 15.<br><br>Brigham, K. & Kumar, B. V. K. V. (2010). Subject identification from electroencephalogram (Eeg) signals during imagined speech, 2010 Fourth Ieee International Conference on Biometrics: Theory, Applications and Systems (Btas), 2010, pp. 1-8.	3820
Metaphorical imagination	The capacity to structure abstract concepts by mapping patterns of bodily experience onto them through metaphor, translating embodied knowledge into linguistic and conceptual meaning	Stephens, V., Matthews, L., Cornelissen, J. P., & Rowlands, H. (2022). Building novel supply chain theory using "metaphorical imagination". Journal of Supply Chain Management, 58(1), 124-139.<br><br>Gibbs, R. W. (2005). Embodiment in metaphorical imagination. Grounding cognition: The role of perception and action in memory, language, and thinking, 65, 92.	4180
Episodic simulation	Ability to imagine events, to simulate perception of events, different people, people that may be real in the world or not, different times, time agnostic events, etc.	Mahr, J. B. (2020). The dimensions of episodic simulation. Cognition, 196, 104085.<br><br>Benoit, R. G., & Schacter, D. L. (2015). Specifying the core network supporting episodic simulation and episodic memory by activation likelihood estimation. Neuropsychologia, 75, 450-457.<br><br>Gaesser, B., & Schacter, D. L. (2014). Episodic simulation and episodic memory can increase intentions to help others. Proceedings of the National Academy of Sciences, 111(12), 4415-4420.	4420
Constructive imagination	Constructive imagination involves multiple processes that allow for engagement with an imagined scenario, invention, interaction, etc.	Van Leeuwen, N. (2013). The meanings of "imagine" part I: Constructive imagination. Philosophy Compass, 8(3), 220-230.	5010
Kinesthetic imagery	Without physically moving, engaging in the sensory experience of performing an action, which can also involve force, effort (regarding muscle movement, balance, and coordination), and spatial sensations	Christensen, J., & Borhani, K. (2020). Dance and the Imagination: Be a Butterfly! In A. Abraham (Ed.), The Cambridge Handbook of the Imagination (Cambridge Handbooks in Psychology, pp. 620-634). Cambridge: Cambridge University Press.<br><br>Di Corrado D, Guarnera M, Vitali F, Quartiroli A, Coco M. (2019). Imagery ability of elite level athletes from individual vs. team and contact vs. no-contact sports. PeerJ.<br><br>Lotze, M. (2013). Kinesthetic imagery of musical performance. Frontiers in human neuroscience, 7, 280.<br><br>Guillot, A., Collet, C., Nguyen, V. A., Malouin, F., Richards, C., & Doyon, J. (2009). Brain activity during visual versus kinesthetic imagery: an fMRI study. Human brain mapping, 30(7), 2157-2172.<br><br>Timothy Raphael, & Joseph Roach. (2009). Activating the Kinesthetic Imagination. Transformations: The Journal of Inclusive Scholarship and Pedagogy, 20(1), 119–130.<br><br>Callow, N., & Waters, A. (2005). The effect of kinesthetic imagery on the sport confidence of flat-race horse jockeys. Psychology of Sport and Exercise, 6(4), 443-459.<br><br>Féry, Y. A. (2003). Differentiating visual and kinesthetic imagery in mental practice. Canadian Journal of Experimental Psychology/Revue canadienne de psychologie expérimentale, 57(1), 1.<br><br>Hall, C., Pongrac, J., & Buckholz, E. (1985). The measurement of imagery ability. Human Movement Science, 4(2), 107–118.	5440

Dimension	Definition	Sample Citations	Google Scholar Hits
Constructive memory	Memory as a creative process rather than an exact recall of past events; memories are influenced by knowledge, emotional state, other information present in working memory, physical location, and so on.	Gaesser, B. (2013). Constructing Memory, Imagination, and Empathy: A Cognitive Neuroscience Perspective. Frontiers in Psychology, 3.<br><br>Schacter, D. L., & Addis, D. R. (2007). The cognitive neuroscience of constructive memory: remembering the past and imagining the future. Philosophical transactions of the Royal Society of London. Series B, Biological sciences, 362(1481), 773–786.<br><br>Schacter, D. L., Norman, K. A., & Koutstaal, W. (1998). The cognitive neuroscience of constructive memory. Annual Review of Psychology, 49(1), 289–318.<br><br>Brewer, W. F., and Treyens, J. C. (1981). Role of schemata in memory for places. Cognitive Psychology, 13(2), 207–230.	6220
Episodic future thinking	The ability to mentally project oneself into the future in order to construct/experience possible future scenarios, rooted in the retrieval of past memories and combining them with possible novel future events	Hollis-Hansen, K., O'Donnell, S. E., Seidman, J. S., Brande, S. J., & Epstein, L. H. (2019). Improvements in episodic future thinking methodology: Establishing a standardized episodic thinking control. PLOS One, 14(3): e0214397.<br><br>Gaesser, B., Keeler, K., & Young, L. (2018). Moral imagination: Facilitating prosocial decision-making through scene imagery and theory of mind. Cognition, 171, 180–193.<br><br>Schacter, D. L., Benoit, R. G., & Szpunar, K. K. (2017). Episodic future thinking: mechanisms and functions. Current opinion in behavioral sciences, 17, 41-50.<br><br>Schacter, D. L., Benoit, R. G., De Brigard, F., & Szpunar, K. K. (2015). Episodic future thinking and episodic counterfactual thinking: Intersections between memory and decisions. Neurobiology of learning and memory, 117, 14-21.<br><br>Schacter, D. L., & Addis, D. R. (2007). The cognitive neuroscience of constructive memory: remembering the past and imagining the future. Philosophical transactions of the Royal Society of London. Series B, Biological sciences, 362(1481), 773–786.<br><br>Atance, C. M., & O'Neill, D. K. (2005). The emergence of episodic future thinking in humans. Learning and motivation, 36(2), 126-144.<br><br>Atance, C. M., & O'Neill, D. K. (2001). Episodic future thinking. Trends in Cognitive Sciences, 5(12), 553-559.	7450
Imaginative Projection/Mindreading	The act of using one's own mental processes, such as beliefs, intentions, and emotions, in order to attribute mental states to others and make behavioral predictions about others or interpret their behavior.	Langland-Hassan, P. (2020). Explaining Imagination. Oxford: Oxford University Press.<br><br>Spaulding, S. (2020). What is mindreading?. Wiley Interdisciplinary Reviews: Cognitive Science, 11(3), e1523.<br><br>Kind, A. (Ed.). (2016). The Routledge Handbook of Philosophy of Imagination (1st ed.). Routledge.<br><br>Carruthers, P. (2013). Mindreading in infancy. Mind & Language, 28(2), 141-172.<br><br>Dimaggio, G., Lysaker, P. H., Carcione, A., Nicolò, G., & Semerari, A. (2008). Know yourself and you shall know the other… to a certain extent: multiple paths of influence of self-reflection on mindreading. Consciousness and cognition, 17(3), 778-789.<br><br>Strauss, C. (2004). Is empathy gendered and, if so, why? An approach from feminist psychological anthropology. Ethos, 32 (4), 432-457.<br><br>Mezirow, J. (1991). Transformation theory and cultural context: A reply to Clark and Wilson. Adult education quarterly, 41 (3), 188-192.	7530
Productive imagination	The capacity to generate genuinely new meanings, structures, or possibilities: not just to recall or re-present what already exists, but to actively synthesize, transform, and invent	Boytsova, J. A., Danko, S. G., & Solovjeva, M. L. (2017). States of productive and reproductive imagination reflected in the local synchronization of alpha and theta Eeg frequencies. Human Physiology, 43(3), 353-357.<br><br>Puente-Díaz, R., & Cavazos-Arroyo, J. (2017). Creative self-efficacy: The influence of affective states and social persuasion as antecedents and imagination and divergent thinking as consequences. Creativity Research Journal, 29(3), 304-312.<br><br>Liang, C., Chang, C. C., & Hsu, Y. (2013). Personality and psychological factors predict imagination: Evidence from Taiwan. Learning and Individual Differences, 27, 67-74.<br><br>Liang, C., Chang, C. C., Chang, Y., & Lin, L. J. (2012). The Exploration of Indicators of Imagination. Turkish Online Journal of Educational Technology-Tojet, 11(3), 366-374.<br><br>Rieber, R. W., & Carton, A. S. (1987). Imagination and its development in childhood. In The collected works of Ls Vygotsky (pp. 339-349) Springer, Boston, Ma.	7630
Musical imagination	The ability to simulate the experience of music (both novel and existing) without the presence of physical music	Martin, S., Mikutta, C., Leonard, M. K., Hungate, D., Koelsch, S., Shamma,S., Chang, E. F., del R Millán, J., Knight, R. T., Pasley, B. N. (2018). Neural Encoding of Auditory Features during Music Perception and Imagery. Cerebral Cortex, 28(12), 4222–4233.<br><br>Lima, C. F., Krishnan, C., Scott, S. K. (2016). Roles of Supplementary Motor Areas in Auditory Processing and Auditory Imagery. Trends in Neurosciences, 39(8), 527-542.<br><br>Zatorre, R. J. & Halpern, A. R. (2005). Mental Concerts: Musical Imagery and Auditory Cortex. Neuron, 47(1), 9-12.<br><br>Brodsky, W., Henik, A., Rubinstein, B. S. et al. (2003). Auditory imagery from musical notation in expert musicians. Perception & Psychophysics 65, 602–612.	7780
Aesthetic engagement	The process of experiencing aesthetics of different modalities (art, music, multi sensory, literary, etc.).	Abraham, A. (2016). The imaginative mind. Human brain mapping, 37(11), 4197-4211.	8790
Exploratory talk	A group discussion wherein shared knowledge construction brought on by constructive criticism, cooperation, counter proposals, and active participation occurs	Webb, P., Whitlow, J. W., & Venter, D. (2017). From exploratory talk to abstract reasoning: A case for far transfer?. Educational psychology review, 29(3), 565-581.<br><br>Knight, S., & Mercer, N. (2015). The role of exploratory talk in classroom search engine tasks. Technology, Pedagogy and Education, 24(3), 303-319.<br><br>Reninger, K. B., & Rehark, L. (2009). Discussions in a fourth-grade classroom: Using exploratory talk to promote children's dialogic identities. Language Arts, 86(4), 268-279.<br><br>Wegerif, R., Mercer, N., & Dawes, L. (1999). From social interaction to individual reasoning: An empirical investigation of a possible socio-cultural model of cognitive development. Learning and instruction, 9(6), 493-516.<br><br>Wegerif, R. (1996). Using computers to help coach exploratory talk across the curriculum. Computers & Education, 26(1-3), 51-60.	9480
Lucid dreaming	Lucid dreaming is a dream where the dreamer is aware they are dreaming.	Ichikawa, J. (2009). Dreaming and imagination. Mind & Language, 24(1), 103-121.<br><br>Schredl, M., & Erlacher, D. (2004). Lucid dreaming frequency and personality. Personality and Individual Differences, 37(7), 1463-1473.<br><br>LaBerge, S. (1990). Lucid dreaming: psychophysiological studies of consciousness during REM sleep.	11000
Hypothetical reasoning	Speculating about possibilities and alternative scenarios and considering their potential implications/consequences	Ball, L. J. (2020). Hypothetical thinking. In A. Abraham (Ed.), The Cambridge handbook of the imagination (pp. 514–528). Cambridge University Press.<br><br>Abraham, A. (2016). The imaginative mind. Human Brain Mapping, 37(11), 4197-4211.<br><br>Davies, J. & Bicknell, J. (2016). Imagination and belief: The microtheories model of hypothetical thinking. Journal of Consciousness Studies, 23(3-4), 31-49.<br><br>De Brigard, F. (2014). Is memory for remembering? Recollection as a form of episodic hypothetical thinking. Synthese, 191, 155–185.<br><br>Ferrante, D., Girotto, V., Stragà, M., & Walsh, C. (2013). Improving the past and the future: A temporal asymmetry in hypothetical thinking. Journal of Experimental Psychology: General, 142(1), 23–27.	13500

Dimension	Definition	Sample Citations	Google Scholar Hits
Mental time travel	Looking back at and reliving past events; simulating/projecting oneself into the future.	Michaelian, K., Perrin, D., Sant'Anna, A., & Schirmer dos Santos, C. (2022). Mental Time Travel. In Glăveanu, V. P. (Ed.), The Palgrave Encyclopedia of the Possible. Palgrave Macmillan, Cham.<br><br>Suddendorf, T., Addis, D. R., & Corballis, M. C. (2009). Mental time travel and the shaping of the human mind. Philosophical Transactions of the Royal Society B, 364, 1317–1324.	15600
Spatial imagery/spatial consciousness	The ability to mentally build, explore, and manipulate representations of space and form — imagining layouts, movement, or arrangements without needing to see them directly. It lets the mind "see" and reason within imagined environments, simulating scenarios, perspectives, and possibilities that aren't physically present. Includes subsets like spatial navigation and scene construction.	Galland, D. and Grønning, M. (2019). Spatial consciousness. The Wiley Blackwell Encyclopedia of Urban and Regional Studies, 1-9.<br><br>El Haj, M., & Lenoble, Q. (2018). Eying the future: Eye movement in past and future thinking. Cortex, 105, 97–103.<br><br>Campos, A. (2013). Reliability and Percentiles of a Measure of Spatial Imagery. Imagination, Cognition and Personality, 32 (4), 427–431.<br><br>Thompson, W. L., Slotnick, S. D., Burrage, M. S., & Kosslyn, S. M. (2009). Two forms of spatial imagery: Neuroimaging evidence. Psychological science, 20(10), 1245-1253.<br><br>Vannucci, M., & Mazzoni, G. (2009). Individual differences in object and spatial imagery: Personality correlates. Personality and Individual Differences, 46(4), 402–405.<br><br>Buckner, R. L., & Carroll, D. C. (2007). Self-projection and the brain. Trends in cognitive sciences, 11(2), 49-57.<br><br>Hassabis, D., & Maguire, E. A. (2007). Deconstructing episodic memory with construction. Trends in cognitive sciences, 11 (7), 299-306.	17200
Verbal imagery/Inner speech	Imagining producing speech or words without vocalizing it; silently articulating a word or sentence	Fernyhough, C., & Borghi, A. M. (2023). Inner speech as language process and cognitive tool. Trends in cognitive sciences, 27 (12), 1180-1193.<br><br>Alderson-Day, B., & Fernyhough, C. (2015). Inner speech: Development, cognitive functions, phenomenology, and neurobiology. Psychological bulletin, 141(5), 931.<br><br>Morin, A. (2005). Possible links between self-awareness and inner speech theoretical background, underlying mechanisms, and empirical evidence. Journal of Consciousness Studies, 12(4-5), 115-134.<br><br>Shergill, S., Bullmore, E., Brammer, M., Williams, S., Murray, R., & McGuire, P. (2001). A functional study of auditory verbal imagery. Psychological Medicine, 31(2), 241-253.<br><br>McGuire, P., Silbersweig, D., Murray, R., David, A., Frackowiak, R., & Frith, C. (1996). Functional anatomy of inner speech and auditory verbal imagery. Psychological Medicine, 26(1), 29-38.	17700
Perspective-taking	Visualizing or conjecturing what it might be like to experience from another's perspective. Related to and often utilized by theory of mind, with the exception that perspective-taking can also occur visually or kinesthetically, without a social or emotional component	Healey, M. L., & Grossman, M. (2018). Cognitive and affective perspective-taking: evidence for shared and dissociable anatomical substrates. Frontiers in neurology, 9, 491.<br><br>Batson, C. D., Early, S., & Salvarani, G. (1997). Perspective taking: Imagining how another feels versus imaging how you would feel. Personality and social psychology bulletin, 23(7), 751-758.	19400
Visual imagination	Using visual imagery, this involves creating visual mental representations, but is a broader constructive capacity or activity that uses such representations to create, interpret, transform, or recombine possibilities	Wang, Y., Li, J., Wang, Z., Liang, B., Jiao, B., Zhang, P., … & Liu, M. (2021). Spontaneous activity in primary visual cortex relates to visual creativity. Frontiers in Human Neuroscience, 15.<br><br>Jankowska, D. M., Gajda, A., & Karwowski, M. (2019). How children's creative visual imagination and creative thinking relate to their representation of space. International Journal of Science Education, 41(8), 1096-1117.<br><br>Jankowska, D. M., & Karwowski, M. (2015). Measuring creative imagery abilities. Frontiers in psychology, 6, 1591.<br><br>Atwood, G. (1971). An experimental study of visual imagination and memory. Cognitive Psychology, 2(3), 290-299.<br><br>Rimm, D. C., & Bottrell, J. (1969). Four measures of visual imagination. Behaviour research and therapy, 7(1), 63-69.	20000
Conceptual blending	The integration of elements from different domains or subject matters to create a novel concept from other concepts.	Friedlander, K. J. (2024). The Psychology of Creative Performance and Expertise. Routledge.<br><br>Fauconnier, G., & Turner, M. (2003). Conceptual blending, form and meaning. Recherches en communication, 19, 57-86.	20700
Counterfactual thinking	Imagining alternative outcomes or scenarios of a past event. Can lead to emotions such as regret, envy or hope.	Kennedy, C., Deane, F. P., Chan, A. Y. C. (2021). "What Might Have Been…": Counterfactual Thinking, Psychological Symptoms and Posttraumatic Growth When a Loved One is Missing. Cognitive Therapy and Research, 45, 322-332.<br><br>Parikh, N, LaBar, K. S., De Brigard, F. (2020). Phenomenology of counterfactual thinking is dampened in anxious individuals. Cognition and Emotion, 34(8), 1737-1745.<br><br>Coricelli, G., & Rustichini, A. (2010). Counterfactual thinking and emotions: regret and envy learning. Philosophical Transactions of the Royal society B: Biological sciences, 365(1538), 241-247.<br><br>Kray, L. J., George, L. G., Liljenquist, K. A., Galinsky, A. D., Tetlock, P. E., & Roese, N. J. (2010). From what might have been to what must have been: Counterfactual thinking creates meaning. Journal of Personality and Social Psychology, 98(1), 106–118.<br><br>Epstude, K., & Roese, N. J. (2008). The Functional Theory of Counterfactual Thinking. Personality and Social Psychology Review, 12(2), 168–192.<br><br>McCloy, R., Byrne, R. M. J. (2000). Counterfactual thinking about controllable events. Memory & Cognition 28, 1071–1078.	22100
False memory	A phenomenon that presents as an accurate memory but is in fact a misremembrance. False memories can occur by malfunction (mistake) or be implanted by another person. This can occur via purposefully or accidentally.	Yu, J., Tao, Q., Zhang, R., Chan, C. C., & Lee, T. M. (2019). Can fMRI discriminate between deception and false memory? A meta-analytic comparison between deception and false memory studies. Neuroscience & Biobehavioral Reviews, 104, 43-55.<br><br>Kaplan, R. L., Van Damme, I., Levine, L. J., & Loftus, E. F. (2016). Emotion and false memory. Emotion Review, 8(1), 8-13.	37200
Social imagination	Mentally representing, simulate, visualizing and reconfiguring social relationships, systems, norms, and collective futures.	Celarent, B. & Abbott, A. (2017). Varieties of Social Imagination. Chicago: University of Chicago Press.<br><br>Hart-Brinson, P. (2016). The social imagination of homosexuality and the rise of same-sex marriage in the united states. Socius: Sociological Research for a Dynamic World, 2, 237802311663055.<br><br>Lysaker, J. T., & Miller, A. (2013). Engaging social imagination: The developmental work of wordless book reading. Journal of Early Childhood Literacy, 13(2), 147-174.<br><br>Vertovec, S. (2012). Diversity and the Social Imaginary. European Journal of Sociology, 53(3), 287-312.	39300
Mind-wandering	When attention strays from a task at hand to an internal experience, world, or imagining.	Villena-Gonzalez, M., & Cosmelli, D. (2020). Imagination and mind wandering: Two sides of the same coin? A brain dynamics perspective. In Creativity and the wandering mind (pp. 93-120). Academic Press.<br><br>Agnoli, S., Vanucci, M., Pelagatti, C., & Corazza, G. E. (2018). Exploring the link between mind wandering, mindfulness, and creativity: A multidimensional approach. Creativity Research Journal, 30(1), 41-53.<br><br>Smallwood, J., & Schooler, J. W. (2015). The science of mind wandering: Empirically navigating the stream of consciousness. Annual review of psychology, 66(1), 487-518.	51300
Pretend play	A type of play in which children engage; spending time with "unreality" in order to learn how to think about the "real" world.	Thompson, B. N. & Goldstein, T. R. (2019). Disentangling pretend play measurement: Defining the essential elements and developmental progression of pretense. Developmental Review, 52, 24-41.<br><br>Gopnik, A., & Walker, C. M. (2013). Considering counterfactuals: The relationship between causal learning and pretend play. American Journal of Play, 6(1), 15-28.	56300
Moral imagination	Considering different actions in ethical situations, understanding their consequences, and empathizing with those affected by them	Werhane, P.H. Moral Imagination and Systems Thinking. Journal of Business Ethics 38, 33–42 (2002).<br><br>Litz, R. (2000). [Review of Moral Imagination and Management Decision Making, by P. C. Werhane]. The Academy of Management Review, 25(1), 256–259.	61300
Divergent thinking	Producing several possible solutions, scenarios, combinations of possibilities, etc.	Benedek, M., & Fink, A. (2019). Toward a neurocognitive framework of creative cognition: the role of memory, attention, and cognitive control. Current Opinion in Behavioral Sciences, 27, 116–122.	84900

Dimension	Definition	Sample Citations	Google Scholar Hits
Mental rotation	The visuospatial ability to rotate a mental image around a chosen axis in 3D space.	Città, G., Gentile, M., Allegra, M., Arrigo, M., Conti, D., Ottaviano, S., & Sciortino, M. (2019). The effects of mental rotation on computational thinking. Computers & Education, 141, 103613.<br><br>Zacks, J. M. (2008). Neuroimaging studies of mental rotation: a meta-analysis and review. Journal of cognitive neuroscience, 20(1), 1-19.<br><br>Parsons, T. D., Larson, P., Kratz, K., Thiebaux, M., Bluestein, B., Buckwalter, J. G., & Rizzo, A. A. (2004). Sex differences in mental rotation and spatial rotation in a virtual environment. Neuropsychologia, 42(4), 555-562.<br><br>Marmor, G. S., & Zaback, L. A. (1976). Mental rotation by the blind: Does mental rotation depend on visual imagery? Journal of Experimental Psychology: Human Perception and Performance, 2(4), 515–521.<br><br>Shepard, R. N., & Metzler, J. (1971). Mental rotation of three-dimensional objects. Science, 171(3972), 701-703.	91500
Daydreaming	Attending to an internal world and is not necessarily influenced by outside stimuli. It can be voluntary or involuntary	Theodor-Katz, N., Somer, E., Hesseg, R. M., & Soffer-Dudek, N. (2022). Could immersive daydreaming underlie a deficit in attention? The prevalence and characteristics of maladaptive daydreaming in individuals with attention-deficit/hyperactivity disorder. Journal of Clinical Psychology, 78(11), 2309-2328.<br><br>West, M. J., & Somer, E. (2020) Empathy, emotion regulation, and creativity in immersive and maladaptive daydreaming. Imagination, Cognition and Personality, 39(4), 358-373.	105000
Creative imagination	The ability to take existing objects, ideas, etc. and generate something novel or an improvement on a previous work. It is also the ability to generate something entirely new.	Meyer, J., Thoma, G. B., Kampschulte, L., & Köller, O. (2023). Openness to experience and museum visits: Intellectual curiosity, aesthetic sensitivity, and creative imagination predict the frequency of visits to different types of museums. Journal of Research in Personality, 103, 104352.<br><br>Hashim, A. T. M., Jamil, M. R. M., & Mamat, N. (2022). Creative Imagination Skills of Preschool Students: Meta Analysis of Antecedent Factors to Encourage Students Achievement. International Journal of Academic Research in Business and Social Sciences, 12(12), 2803-2811.<br><br>Karwowski, M. (2008). Measuring creativity using the Test of Creative Imagination (Tci). Part 1. Presentation of a new instrument to measure creative potential. The New Educational Review, 14(1), 44-53.<br><br>Karwowski, M. (2008). Measuring Creativity Using the Test of Creative Imagination (Tci). Part 2. On Validity of the Tci. The New Educational Review, 15(2), 216-231.	107000
Motor cognition/Motor imagery	A mental performance of a physical movement.	Savaki, H. E., & Raos, V. (2019). Action Perception and Motor Imagery: Mental Practice of Action. Progress in Neurobiology.<br><br>Kim, Y. K., Park E., Lee A., Im, C-H., Kim, Y-H. (2018). Changes in network connectivity during motor imagery and execution. PLoS One. 13(1). e0190715.<br><br>O'Shea, H. & Moran, A. (2017). Does Motor Simulation Theory Explain the Cognitive Mechanisms Underlying Motor Imagery? A Critical Review. Frontiers in Human Neuroscience, 17.<br><br>Pulvermüller, F., Moseley, R. L., Egorova, N., Shebani, Z., & Boulenger, V. (2014). Motor cognition–motor semantics: Action perception theory of cognition and communication. Neuropsychologia, 55, 71-84.<br><br>Hétu, S., Grégoire, M., Saimpont, A., Coll, M. P., Eugène, F., Michon, P. E., Jackson, P. L. (2013). The neural network of motor imagery: An Ale meta-analysis. Neuroscience & Biobehavioral Reviews, 37(5), 930-949.<br><br>Gallese, V., Rochat, M., Cossu, G., Sinigaglia, C. (2009). Motor cognition and its role in the phylogeny and ontogeny of action understanding. Developmental Psychology, 45(1), 103-113.<br><br>Fuentes, C. T., & Bastian, A. J. (2007). 'Motor cognition'—what is it and is the cerebellum involved?. The Cerebellum, 6(3), 232-236.<br><br>Sommerville, J. A., & Decety, J. (2006). Weaving the fabric of social interaction: Articulating developmental psychology and cognitive neuroscience in the domain of motor cognition. Psychonomic Bulletin & Review, 13(2), 179-200.<br><br>Lotze, M. & Halsband, U.(2006). Motor imagery. Journal of Physiology-Paris, 99(4-6), 386-395.<br><br>Jackson, P. L., & Decety, J. (2004). Motor cognition: A new paradigm to study self–other interactions. Current opinion in Neurobiology, 14(2), 259-263.	202000
Visual imagery	The act of creating mental images or pictures in one's mind without physical external stimuli.	Currie, G. (1995). Visual imagery as the simulation of vision. Mind & Language, 10(1-2), 25–44.<br><br>Roland, P. E., & Gulyás, B. (1994). Visual imagery and visual representation. Trends in neurosciences, 17(7), 281–297.	280000
Mental imagery	The experience of sensory information without external stimuli	Pearson, J. (2019). The human imagination: the cognitive neuroscience of visual mental imagery. Nature reviews neuroscience, 20(10), 624-634.	332000
Process simulation	A type of mental simulation wherein one imagines a step-by-step plan to achieve a certain goal or complete a certain activity	Prokopowicz, A., & Byrka, K. (2021). Effectiveness of mental simulations on the early mobilization of patients after cesarean section: a randomized controlled trial. Scientific reports, 11(1), 1-8.<br><br>Li, J., Yang, R., Cui, J., & Guo, Y. (2019). Imagination matters when you shop online: The moderating role of mental simulation between materialism and online impulsive buying. Psychology Research and Behavior Management, 12, 1071.<br><br>Petrova, P. K., & Cialdini, R. B. (2018). Evoking the imagination as a strategy of influence. In Handbook of consumer psychology (pp. 510-528). Routledge.	355000
Mental simulation	The processes, abilities, and/or skills that allow us to create imagined visual images, motor actions, narratives of stories, and generate possible futures	Hamrick, J. B. (2019). Analogues of mental simulation and imagination in deep learning. Current Opinion in Behavioral Sciences, 29, 8-16.<br><br>Li, J., Yang, R., Cui, J., & Guo, Y. (2019). Imagination matters when you shop online: The moderating role of mental simulation between materialism and online impulsive buying. Psychology Research and Behavior Management, 1071-1079.	456000
Dreaming	A subjective experience that occurs when asleep. It can involve all the senses, a range of narrative complexity, and be remembered or forgotten upon awakening.	Siclari, F., Baird, B., Perogamvros, L., Bernardi, G., LaRocque, J. J., Riedner, B., ... & Tononi, G. (2017). The neural correlates of dreaming. Nature neuroscience, 20(6), 872-878.<br><br>Revonsuo, A. (2000). The reinterpretation of dreams: An evolutionary hypothesis of the function of dreaming. Behavioral and brain sciences, 23(6), 877-901.	814000
Creative thinking	The ability to produce novel ideas and unique solutions to problems to generate a new or remodeled physical or conceptual item or work	Gafour, O. W., & Gafour, W. A. (2020). Creative thinking skills–A review article. Journal of Education and e-Learning, 4(1), 44-58.<br><br>Beaty, R. E., Benedek, M., Silvia, P. J., & Schacter, D. L. (2016). Creative cognition and brain network dynamics. Trends in Cognitive Sciences, 20(2), 87–95.<br><br>Runco, M. A., & Jaeger, G. J. (2012). The standard definition of creativity. Creativity Research Journal, 24(1), 92–96.	824000
Theory of mind	The ability to attribute and understand others' mental states, recognizing that individuals may hold differing perspectives and beliefs from one's own.	Braunsdorf, M., Freches, G. B., Roumazeilles, L., Eichert, N., Schurz, M., Uithol, S., Bryant, K. L., Mars, R. B.(2021). Does the temporal cortex make us human? A review of structural and functional diversity of the primate temporal lobe. Neuroscience & Biobehavioral Reviews, 131, 400-410.<br><br>Schurz, M., Radua, J., Tholen, M. G., Maliske, L., Margulies, D. S., Mars, R. B., Sallet, J., & Kanske, P. (2021). Toward a hierarchical model of social cognition: A neuroimaging meta-analysis and integrative review of empathy and theory of mind. Psychological Bulletin, 147(3), 293–327.<br><br>Yuk, V., Urbain, C., Pang, E. W., Anagnostou, E., Buchsbaum, D., Taylor, M. J. (2018). Do you know what I'm thinking? Temporal and spatial brain activity during a theory-of-mind task in children with autism. Developmental Cognitive Neuroscience, 34, 139-147.<br><br>Schaafsma, S. M., Pfaff, D. W., Spunt, R. P., Adolphs, R. (2015). Deconstructing and reconstructing theory of mind. Trends in Cognitive Sciences, 19(2), 65-72.<br><br>Schurz, M., Radua, J., Aichhorn, M., Richlan, F., Perner, J. (2014). Fractionating theory of mind: A meta-analysis of functional brain imaging studies. Neuroscience & Biobehavioral Reviews, 42, 9-34.	833000
Empathy	Mentally placing yourself in another's situation and simulating their thoughts and feelings by taking on their perspectives.	Cuff, B. M. P., Brown, S. J., Taylor, L., & Howat, D. J. (2016). Empathy: A Review of the Concept. Emotion Review, 8(2), 144–153.<br><br>Eisenberg, N., & Strayer, J. (Eds.). (1987). Empathy and its development. Cambridge University Press.	2860000
