# Supplementary material for: Defining imagination: a conceptual mapping and provisional model
Source: Front Psychol. 2026 Sep 21;17:1888826. doi: 10.3389/fpsyg.2026.1888826 (PMC13639878; doi:10.3389/fpsyg.2026.1888826)
Supplement: Supplementary file 2 [file Data_Sheet_2.pdf]

Dimension	Working Definition	Example	Representative Citations	Google Scholar "Hits"
**Cross-Domain**				
Dreaming	A subjective experience that occurs when asleep. It can involve all the senses, a range of narrative complexity, and be remembered or forgotten upon awakening	While sleeping, you participate in an elaborate story of living in a castle. You are a knight and have to contend with a neighboring kingdom applying pressure to the Southern border. You balance preparing your army with being in love with the princess	Siclari, F., Baird, B., Perogamvros, L., Bernardi, G., LaRocque, J. J., Riedner, B.,... & Tononi, G. (2017). The neural correlates of dreaming. Nature neuroscience, 20(6), 872-878.<br><br>Revonsuo, A. (2000). The reinterpretation of dreams: An evolutionary hypothesis of the function of dreaming. Behavioral and brain sciences, 23(6), 877-901.	814000
Mental simulation	The processes, abilities, and/or skills that allow us to create imagined visual images, motor actions, narratives of stories, and generate possible futures	You imagine the discussion you are going to have with your boss later to negotiate a pay raise. You mentally rehearse your pitch, and also include the confidence with which you want to approach your boss. You have a strong but positive tone of voice and a firm handshake. Additionally, you imagine what your boss might say or think in response to your speech so you can best prepare yourself	Hamrick, J. B. (2019). Analogues of mental simulation and imagination in deep learning. Current Opinion in Behavioral Sciences, 29, 8-16.<br><br>Li, J., Yang, R., Cui, J., & Guo, Y. (2019). Imagination matters when you shop online: The moderating role of mental simulation between materialism and online impulsive buying. Psychology Research and Behavior Management, 1071-1079.	456000
Mental imagery	The experience of sensory information without external stimuli	You imagine yourself walking through the rain in order to reach an underground cavern. On the way, you can see and feel the cold droplets hitting you and the wind pushing against you, your clothes wrapping around your body, even though in reality you are dry and indoors	Pearson, J. (2019). The human imagination: the cognitive neuroscience of visual mental imagery. Nature reviews neuroscience, 20(10), 624-634.	332000
Creative imagination	The ability to take existing objects, ideas, etc. and generate something novel or an improvement on a previous work. It is also the ability to generate something entirely new	As you sit in an art class and contemplate the composition of your next art piece, you imagine a scene in which you are up in the sky, flying across the clouds	Meyer, J., Thoma, G. B., Kampschulte, L., & Köller, O. (2023). Openness to experience and museum visits: Intellectual curiosity, aesthetic sensitivity, and creative imagination predict the frequency of visits to different types of museums. Journal of Research in Personality, 103, 104352.<br><br>Hashim, A. T. M., Jamil, M. R. M., & Mamat, N. (2022). Creative Imagination Skills of Preschool Students: Meta Analysis of Antecedent Factors to Encourage Students Achievement. International Journal of Academic Research in Business and Social Sciences, 12(12), 2803-2811.<br><br>Karwowski, M. (2008). Measuring creativity using the Test of Creative Imagination (Tci). Part 1. Presentation of a new instrument to measure creative potential. The New Educational Review, 14(1), 44-53.<br><br>Karwowski, M. (2008). Measuring Creativity Using the Test of Creative Imagination (Tci). Part 2. On Validity of the Tci. The New Educational Review, 15(2), 216-231.	107000
Mind-wandering	When attention strays from a task at hand to an internal experience, world, or imagining	You sit at your computer working on a paper. While you try to think of ways to strengthen your argument, you remember the trip you are supposed to take to Colorado next week. Suddenly, you are thinking of a documentary you recently watched about a cult in Colorado. Then, you are considering how upbringing influences your personality and vulnerability to manipulation and wonder if you could be convinced to join a cult	Villena-Gonzalez, M., & Cosmelli, D. (2020). Imagination and mind wandering: Two sides of the same coin? A brain dynamics perspective. In Creativity and the wandering mind (pp. 93-120). Academic Press.<br><br>Agnoli, S., Vanucci, M., Pelagatti, C., & Corazza, G. E. (2018). Exploring the link between mind wandering, mindfulness, and creativity: A multidimensional approach. Creativity Research Journal, 30(1), 41-53.<br><br>Smallwood, J., & Schooler, J. W. (2015). The science of mind wandering: Empirically navigating the stream of consciousness. Annual review of psychology, 66(1), 487-518.	51300
Lucid dreaming	Lucid dreaming is a dream where the dreamer is aware they are dreaming.	Immersed in a dream wherein you are swimming at the beach with friends, you become aware that you are dreaming and that what you are experiencing is not reality. You decide to turn into a mermaid and explore an underwater city	Ichikawa, J. (2009). Dreaming and imagination. Mind & Language, 24(1), 103-121.<br><br>Schredl, M., & Erlacher, D. (2004). Lucid dreaming frequency and personality. Personality and Individual Differences, 37(7), 1463-1473.<br><br>LaBerge, S. (1990). Lucid dreaming: psychophysiological studies of consciousness during REM sleep.	11000
Daydreaming	Attending to an internal world and is not necessarily influenced by outside stimuli. It can be voluntary or involuntary	In the middle of a lecture, you begin thinking about an upcoming birthday party and everything else falls away as you focus solely on the events that might occur at the party and who you hope to see there	Theodor-Katz, N., Somer, E., Hesseg, R. M., & Soffer-Dudek, N. (2022). Could immersive daydreaming underlie a deficit in attention? The prevalence and characteristics of maladaptive daydreaming in individuals with attention-deficit/hyperactivity disorder. Journal of Clinical Psychology, 78(11), 2309-2328.<br><br>West, M. J., & Somer, E. (2020) Empathy, emotion regulation, and creativity in immersive and maladaptive daydreaming. Imagination, Cognition and Personality, 39(4), 358-373.	105000
Aesthetic engagement	The process of experiencing aesthetics of different modalities (art, music, multi sensory, literary, etc.).	As you read a hauntingly descriptive novel, you become fully immersed as your mind vividly populates the characters' emotions, voices, and physical surroundings	Abraham, A. (2016). The imaginative mind. Human brain mapping, 37 (11), 4197-4211.	87
Constructive imagination	Constructive imagination involves multiple processes that allow for engagement with an imagined scenario, invention, interaction, etc	You are an FBI investigator working a case. You have a number of key pieces of evidence, but cannot figure out how they fit together. You imagine different ways that the evidence and known events piece together, allowing you to unlock the case	Van Leeuwen, N. (2013). The meanings of "imagine" part I: Constructive imagination. Philosophy Compass, 8(3), 220-230.	5010
Propositional imagination	Accepting that a certain made-up situation or set of circumstances is actually the case, typically used in mental exercises, such as thought experiments	When reading a book, you accept that there is a young man named Romeo and a young woman named Juliet from rival families that meet and quickly fall in love, despite knowing that these individuals are fictional	Murphy, A. (2022). Imagination in science. Philosophy Compass, 17(6), e12836.<br><br>Nichols, S. (2019). The propositional imagination. In The Routledge Companion to Philosophy of Psychology (pp. 360-369). Routledge.	385
**Cognitive-Narrative**				
Creative thinking	The ability to produce novel ideas and unique solutions to problems to generate a new or remodeled physical or conceptual item or work	An artwork (visual, aural, mixed, literary, etc.) or an improved method for powering vehicles; the ability to think outside the box. Or, on a rainy day, you find yourself without an umbrella so you imagine different alternative ways to stay dry	Gafour, O. W., & Gafour, W. A. (2020). Creative thinking skills–A review article. Journal of Education and e-Learning, 4(1), 44-58.<br><br>Beaty, R. E., Benedek, M., Silvia, P. J., & Schacter, D. L. (2016). Creative cognition and brain network dynamics. Trends in Cognitive Sciences, 20 (2), 87–95.<br><br>Runco, M. A., & Jaeger, G. J. (2012). The standard definition of creativity. Creativity Research Journal, 24(1), 92–96.	824000
Process simulation	A type of mental simulation wherein one imagines a step-by-step plan to achieve a certain goal or complete a certain activity	As you plan baking a cake for your friend's birthday, you imagine which ingredients will be added first, when to mix the wet and dry ingredients, how long you will need to bake it, when and what to add later, etc	Prokopowicz, A., & Byrka, K. (2021). Effectiveness of mental simulations on the early mobilization of patients after cesarean section: a randomized controlled trial. Scientific reports, 11(1), 1-8.<br><br>Li, J., Yang, R., Cui, J., & Guo, Y. (2019). Imagination matters when you shop online: The moderating role of mental simulation between materialism and online impulsive buying. Psychology Research and Behavior Management, 12, 1071.<br><br>Petrova, P. K., & Cialdini, R. B. (2018). Evoking the imagination as a strategy of influence. In Handbook of consumer psychology (pp. 510-528). Routledge.	355000
Divergent thinking	Producing several possible solutions, scenarios, combinations of possibilities, etc	You lost your umbrella but it is raining outside and you need to go get lunch. So, you imagine alternative ways to make it to your car while staying dry without an umbrella	Benedek, M., & Fink, A. (2019). Toward a neurocognitive framework of creative cognition: the role of memory, attention, and cognitive control. Current Opinion in Behavioral Sciences, 27, 116–122.	84900

Dimension	Working Definition	Example	Representative Citations	Google Scholar "Hits"
Counterfactual thinking	Imagining alternative outcomes or scenarios of a past event. Can lead to emotions such as regret, envy or hope.	Imagine how your college age son's life would have been different if there had been no COVID outbreak in 2020: how their Zoom graduation could have been different, or their first year of college not having been on Zoom	Kennedy, C., Deane, F. P., Chan, A. Y. C. (2021). "What Might Have Been…": Counterfactual Thinking, Psychological Symptoms and Posttraumatic Growth When a Loved One is Missing. Cognitive Therapy and Research, 45, 322-332.<br><br>Parikh, N, LaBar, K. S., De Brigard, F. (2020). Phenomenology of counterfactual thinking is dampened in anxious individuals. Cognition and Emotion, 34(8), 1737-1745.<br><br>Coricelli, G., & Rustichini, A. (2010). Counterfactual thinking and emotions: regret and envy learning. Philosophical Transactions of the Royal society B: Biological sciences, 365(1538), 241-247.<br><br>Kray, L. J., George, L. G., Liljenquist, K. A., Galinsky, A. D., Tetlock, P. E., & Roese, N. J. (2010). From what might have been to what must have been: Counterfactual thinking creates meaning. Journal of Personality and Social Psychology, 98(1), 106–118.<br><br>Epstude, K., & Roese, N. J. (2008). The Functional Theory of Counterfactual Thinking. Personality and Social Psychology Review, 12 (2), 168–192.<br><br>McCloy, R., Byrne, R. M. J. (2000). Counterfactual thinking about controllable events. Memory & Cognition 28, 1071–1078.	22100
Conceptual blending	The integration of elements from different domains or subject matters to create a novel concept from other concepts	Describing a slow-moving bureaucratic process as a 'soupy delay' blends the sensory qualities of soup (thick, viscous, resistant) with the abstract experience of waiting, producing an emergent sense of murky sluggishness that is present in neither 'soup' nor 'delay' alone, and that must be actively constructed rather than retrieved from familiar usage.	Friedlander, K. J. (2024). The Psychology of Creative Performance and Expertise. Routledge.<br><br>Fauconnier, G., & Turner, M. (2003). Conceptual blending, form and meaning. Recherches en communication, 19, 57-86.	20700
Hypothetical reasoning	Speculating about possibilities and alternative scenarios and considering their potential implications/consequences	You know that access to drinkable freshwater is a significant problem. You wonder if desalinating ocean water could create a source of fresh water for everyone, and contemplate how to make such a resource available to the communities in need	Ball, L. J. (2020). Hypothetical thinking. In A. Abraham (Ed.), The Cambridge handbook of the imagination (pp. 514–528). Cambridge University Press.<br><br>Abraham, A. (2016). The imaginative mind. Human Brain Mapping, 37 (11), 4197-4211.<br><br>Davies, J. & Bicknell, J. (2016). Imagination and belief: The microtheories model of hypothetical thinking. Journal of Consciousness Studies, 23(3-4), 31-49.<br><br>De Brigard, F. (2014). Is memory for remembering? Recollection as a form of episodic hypothetical thinking. Synthese, 191, 155–185.<br><br>Ferrante, D., Girotto, V., Stragà, M., & Walsh, C. (2013). Improving the past and the future: A temporal asymmetry in hypothetical thinking. Journal of Experimental Psychology: General, 142(1), 23–27.	13500
Productive imagination	The capacity to generate genuinely new meanings, structures, or possibilities: not just to recall or re-present what already exists, but to actively synthesize, transform, and invent	Biologists represent evolution as a tree with branches to help people understand common ancestry and "see" divergence and relatedness	Boytsova, J. A., Danko, S. G., & Solovjeva, M. L. (2017). States of productive and reproductive imagination reflected in the local synchronization of alpha and theta Eeg frequencies. Human Physiology, 43(3), 353-357.<br><br>Puente-Díaz, R., & Cavazos-Arroyo, J. (2017). Creative self-efficacy: The influence of affective states and social persuasion as antecedents and imagination and divergent thinking as consequences. Creativity Research Journal, 29(3), 304-312.<br><br>Liang, C., Chang, C. C., & Hsu, Y. (2013). Personality and psychological factors predict imagination: Evidence from Taiwan. Learning and Individual Differences, 27, 67-74.<br><br>Liang, C., Chang, C. C., Chang, Y., & Lin, L. J. (2012). The Exploration of Indicators of Imagination. Turkish Online Journal of Educational Technology-Tojet, 11(3), 366-374.<br><br>Rieber, R. W., & Carton, A. S. (1987). Imagination and its development in childhood. In The collected works of Ls Vygotsky (pp. 339-349) Springer, Boston, Ma.	7630
Outcome simulation	Contemplating various results and implications of an event, both positive and negative.	Following an interview with a company for your dream position, you wonder what it would be like to get the job (upward outcome simulation) or to get rejected (downward outcome simulation)	Prokopowicz, A., & Byrka, K. (2021). Effectiveness of mental simulations on the early mobilization of patients after cesarean section: a randomized controlled trial. Scientific reports, 11(1), 1-8.<br><br>Li, J., Yang, R., Cui, J., & Guo, Y. (2019). Imagination matters when you shop online: The moderating role of mental simulation between materialism and online impulsive buying. Psychology Research and Behavior Management, 12, 1071.<br><br>Sanna, L. J. (2000). Mental simulation, affect, and personality: A conceptual framework. Current Directions in Psychological Science, 9(5), 168-173.	1360
Goal imagery	A mental experience of attaining a personal goal	You imagine holding the soccer championship trophy after making the winning shot to the goal. Your teammates surround you and you all cheer for your win together	Williams, S. E., & Cumming, J. (2016). Athlete imagery ability: A predictor of confidence and anxiety intensity and direction. International Journal of Sport and Exercise Psychology, 14(3), 268-280.<br><br>Chan, C. K., & Cameron, L. D. (2012). Promoting physical activity with goal-oriented mental imagery: a randomized controlled trial. Journal of Behavioral Medicine, 35(3), 347-363.<br><br>Langens, T. A. (2003). Potential costs of goal imagery: The moderating role of fear of failure. Imagination, Cognition and Personality, 23(1), 27-44.<br><br>Schultheiss, O. C., & Brunstein, J. C. (1999). Goal imagery: Bridging the gap between implicit motives and explicit goals. Journal of personality, 67(1), 1-38.	617
**Cognitive/Narrative - Social/Emotional**				
Empathy	Mentally placing yourself in another's situation and simulating their thoughts and feelings by taking on their perspectives	Imagine your best friend has just lost their job and what they must be feeling in order to truly understand their feelings and offer them support	Cuff, B. M. P., Brown, S. J., Taylor, L., & Howat, D. J. (2016). Empathy: A Review of the Concept. Emotion Review, 8(2), 144–153.<br><br>Eisenberg, N., & Strayer, J. (Eds.). (1987). Empathy and its development. Cambridge University Press.	2860000
Moral imagination	Considering different actions in ethical situations, understanding their consequences, and empathizing with those affected by them	You witness someone striking a child in a supermarket and are overcome with mental scenarios as to how you should react to this situation; should you intervene, should you say something, is it your place to even do anything, etc	Werhane, P.H. Moral Imagination and Systems Thinking. Journal of Business Ethics 38, 33–42 (2002).<br><br>Litz, R. (2000). [Review of Moral Imagination and Management Decision Making, by P. C. Werhane]. The Academy of Management Review, 25(1), 256–259.	61300

Dimension	Working Definition	Example	Representative Citations	Google Scholar "Hits"
Pretend play	A type of play in which children engage; spending time with "unreality" in order to learn how to think about the "real" world	You are four years old and playing with your friends at preschool. You have arranged a pile of cushions to be your "home" and assigned family roles as you play house. Right now you are all working to cook dinner using crayons as your cooking utensils	Thompson, B. N. & Goldstein, T. R. (2019). Disentangling pretend play measurement: Defining the essential elements and developmental progression of pretense. Developmental Review, 52, 24-41.<br><br>Gopnik, A., & Walker, C. M. (2013). Considering counterfactuals: The relationship between causal learning and pretend play. American Journal of Play, 6(1), 15-28.	56300
False memory	A phenomenon that presents as an accurate memory but is in fact a misremembrance. False memories can occur by malfunction (mistake) or be implanted by another person. This can occur via purposefully or accidentally	You search for your keys in the small pocket of your backpack, distinctly remembering placing them there earlier. Shaking your head in disbelief, you later find them in the side pocket where you never place your keys	Yu, J., Tao, Q., Zhang, R., Chan, C. C., & Lee, T. M. (2019). Can fMRI discriminate between deception and false memory? A meta-analytic comparison between deception and false memory studies. Neuroscience & Biobehavioral Reviews, 104, 43-55.<br><br>Kaplan, R. L., Van Damme, I., Levine, L. J., & Loftus, E. F. (2016). Emotion and false memory. Emotion Review, 8(1), 8-13.	37200
Imaginative projection/Mindreading	The act of using one's own mental processes in order to attribute mental states to others and make behavioral predictions about others	You see your friend walk in with a sad expression. You know she had an important job interview earlier in the day, and based on her expression and her general gloomy appearance you can infer that the interview did not go well and she is mentally fixating on what went wrong. You know when she gets sad she likes to rewatch her favorite movie, and you imagine that is what she will be doing later today	Langland-Hassan, P. (2020). Explaining Imagination. Oxford: Oxford University Press.<br><br>Spaulding, S. (2020). What is mindreading?. Wiley Interdisciplinary Reviews: Cognitive Science, 11(3), e1523.<br><br>Kind, A. (Ed.). (2016). The Routledge Handbook of Philosophy of Imagination (1st ed.). Routledge.<br><br>Carruthers, P. (2013). Mindreading in infancy. Mind & Language, 28(2), 141-172.<br><br>Dimaggio, G., Lysaker, P. H., Carcione, A., Nicolò, G., & Semerari, A. (2008). Know yourself and you shall know the other… to a certain extent: multiple paths of influence of self-reflection on mindreading. Consciousness and cognition, 17(3), 778-789.<br><br>Strauss, C. (2004). Is empathy gendered and, if so, why? An approach from feminist psychological anthropology. Ethos, 32(4), 432-457.<br><br>Mezirow, J. (1991). Transformation theory and cultural context: A reply to Clark and Wilson. Adult education quarterly, 41(3), 188-192.	25800
Mental time travel	Looking back at and reliving past events; simulating/projecting oneself into the future	You reminisce about your middle school days and the hallways and classrooms you used to be in every day	Michaelian, K., Perrin, D., Sant'Anna, A., & Schirmer dos Santos, C. (2022). Mental Time Travel. In Glǎveanu, V. P. (Ed.), The Palgrave Encyclopedia of the Possible. Palgrave Macmillan, Cham.<br><br>Suddendorf, T., Addis, D. R., & Corballis, M. C. (2009). Mental time travel and the shaping of the human mind. Philosophical Transactions of the Royal Society B, 364, 1317–1324.	15600
Exploratory talk	A group discussion wherein shared knowledge construction brought on by constructive criticism, cooperation, counter proposals, and active participation occurs	You are working with a team of researchers trying to create solutions to help address the effects of climate change on food scarcity. It's a complicated topic, but by talking through the process with the other researchers and continually giving constructive feedback and counterproposals, you are able to explore solutions.	Webb, P., Whitlow, J. W., & Venter, D. (2017). From exploratory talk to abstract reasoning: A case for far transfer?. Educational psychology review, 29(3), 565-581.<br><br>Knight, S., & Mercer, N. (2015). The role of exploratory talk in classroom search engine tasks. Technology, Pedagogy and Education, 24(3), 303-319.<br><br>Reninger, K. B., & Rehark, L. (2009). Discussions in a fourth-grade classroom: Using exploratory talk to promote children's dialogic identities. Language Arts, 86(4), 268-279.<br><br>Wegerif, R., Mercer, N., & Dawes, L. (1999). From social interaction to individual reasoning: An empirical investigation of a possible socio-cultural model of cognitive development. Learning and instruction, 9(6), 493-516.<br><br>Wegerif, R. (1996). Using computers to help coach exploratory talk across the curriculum. Computers & Education, 26(1-3), 51-60.	9480
Episodic future thinking	The ability to mentally project oneself into the future in order to construct/experience possible future scenarios, rooted in the retrieval of past memories and combining them with possible novel future events	You consider a future vacation, including where you would go, how you would get there, the length of time you would go for, and where you would stay	Hollis-Hansen, K., O'Donnell, S. E., Seidman, J. S., Brande, S. J., & Epstein, L. H. (2019). Improvements in episodic future thinking methodology: Establishing a standardized episodic thinking control. PLOS One, 14(3): e0214397.<br><br>Gaesser, B., Keeler, K., & Young, L. (2018). Moral imagination: Facilitating prosocial decision-making through scene imagery and theory of mind. Cognition, 171, 180–193.<br><br>Schacter, D. L., Benoit, R. G., & Szpunar, K. K. (2017). Episodic future thinking: mechanisms and functions. Current opinion in behavioral sciences, 17, 41-50.<br><br>Schacter, D. L., Benoit, R. G., De Brigard, F., & Szpunar, K. K. (2015). Episodic future thinking and episodic counterfactual thinking: Intersections between memory and decisions. Neurobiology of learning and memory, 117, 14-21.<br><br>Schacter, D. L., & Addis, D. R. (2007). The cognitive neuroscience of constructive memory: remembering the past and imagining the future. Philosophical transactions of the Royal Society of London. Series B, Biological sciences, 362(1481), 773–786.<br><br>Atance, C. M., & O'Neill, D. K. (2005). The emergence of episodic future thinking in humans. Learning and motivation, 36(2), 126-144.<br><br>Atance, C. M., & O'Neill, D. K. (2001). Episodic future thinking. Trends in Cognitive Sciences, 5(12), 553-559.	7450
Constructive memory	Memory as a creative process rather than an exact recall of past events; memories are influenced by knowledge, emotional state, other information present in working memory, physical location, and so on.	Recalling a doctor's waiting room where you sat for twenty minutes last week, you're confident there was a stack of magazines on the side table, though in fact the table was empty. Your memory filled in a detail it considered a typical feature of waiting rooms, without your having noticed the substitution or intending to alter anything.	Gaesser, B. (2013). Constructing Memory, Imagination, and Empathy: A Cognitive Neuroscience Perspective. Frontiers in Psychology, 3.<br><br>Schacter, D. L., & Addis, D. R. (2007). The cognitive neuroscience of constructive memory: remembering the past and imagining the future. Philosophical transactions of the Royal Society of London. Series B, Biological sciences, 362(1481), 773–786.<br><br>Schacter, D. L., Norman, K. A., & Koutstaal, W. (1998). The cognitive neuroscience of constructive memory. Annual Review of Psychology, 49 (1), 289–318.<br><br>Brewer, W. F., and Treyens, J. C. (1981). Role of schemata in memory for places. Cognitive Psychology, 13(2), 207–230.	6220

Dimension	Working Definition	Example	Representative Citations	Google Scholar "Hits"
Episodic simulation	Ability to imagine events, to simulate perception of events, different people, people that may be real in the world or not, different times, time agnostic events, etc	You imagine encountering a unicorn with a shining black coat slowly approaching you on a deserted, alien beach. Its hooves thump softly through the sand and all you hear is the wind, the exhale of the waves, and the warm breath of this magnificent being	Mahr, J. B. (2020). The dimensions of episodic simulation. Cognition, 196, 104085.<br><br>Benoit, R. G., & Schacter, D. L. (2015). Specifying the core network supporting episodic simulation and episodic memory by activation likelihood estimation. Neuropsychologia, 75, 450-457.<br><br>Gaesser, B., & Schacter, D. L. (2014). Episodic simulation and episodic memory can increase intentions to help others. Proceedings of the National Academy of Sciences, 111(12), 4415-4420.	4420
Reproductive imagination	Remembrances; recalling things or events encountered before, like a memory or semantic knowledge. Creating mental images and models from communication with others, reading a book, etc	While at work, you imagine your drive home, visualizing the route and usual sites/landmarks seen on the way	Boytsova, J. A., Danko, S. G., & Solovjeva, M. L. (2017). States of productive and reproductive imagination reflected in the local synchronization of alpha and theta Eeg frequencies. Human Physiology, 43(3), 353-357.<br><br>Liang, C., Chang, C. C., & Hsu, Y. (2013). Personality and psychological factors predict imagination: Evidence from Taiwan. Learning and Individual Differences, 27, 67-74.<br><br>Liang, C., Chang, C. C., Chang, Y., & Lin, L. J. (2012). The Exploration of Indicators of Imagination. Turkish Online Journal of Educational Technology-Tojet, 11(3), 366-374.	2930
Recreative imagination	Re-creating past perceptual experience in the absence of the stimulus. Rather than "reproducing" a mental photograph, the mind is re-creating an experience in the present	An athlete mentally rehearsing a familiar movement sequence or a grieving person vividly recalling the voice or gestures of a loved one	Arcangeli, M. (2022). Chapter: The creative side of recreative imagination; In: The Philosophy of Fiction. Isbn: 9781003139720.<br><br>Langland-Hassan, P. (2020). Explaining Imagination. Oxford: Oxford University Press.<br><br>Kind, A. (Ed.). (2016). The Routledge Handbook of Philosophy of Imagination (1st ed.). Routledge.<br><br>Currie, G., & Ravenscroft, I. (2002). Recreative minds: Imagination in philosophy and psychology. Oup Oxford.	343
Functional imagination	To simulate experiences internally without physical actions in order to predict the benefit or consequence in the real world	You imagine different vacation destinations and mentally explore the benefits of each one before deciding where to book your next trip	Marques, H. G., & Holland, O. (2009). Architectures for functional imagination. Neurocomputing, 72(4-6), 743-759.	168
**Social-Emotional**				
Theory of mind	The ability to generate and experience the mental states, beliefs, emotions, and perspectives of others.	You see what appears to be a mother frantically searching for her child in a shopping mall. You can imagine her panic and urgency, and may even experience some of it yourself, though the situation is not directly affecting you	Braunsdorf, M., Freches, G. B., Roumazeilles, L., Eichert, N., Schurz, M., Uithol, S., Bryant, K. L., Mars, R. B.(2021). Does the temporal cortex make us human? A review of structural and functional diversity of the primate temporal lobe. Neuroscience & Biobehavioral Reviews, 131, 400-410.<br><br>Schurz, M., Radua, J., Tholen, M. G., Maliske, L., Margulies, D. S., Mars, R. B., Sallet, J., & Kanske, P. (2021). Toward a hierarchical model of social cognition: A neuroimaging meta-analysis and integrative review of empathy and theory of mind. Psychological Bulletin, 147(3), 293–327.<br><br>Yuk, V., Urbain, C., Pang, E. W., Anagnostou, E., Buchsbaum, D., Taylor, M. J. (2018). Do you know what I'm thinking? Temporal and spatial brain activity during a theory-of-mind task in children with autism. Developmental Cognitive Neuroscience, 34, 139-147.<br><br>Schaafsma, S. M., Pfaff, D. W., Spunt, R. P., Adolphs, R. (2015). Deconstructing and reconstructing theory of mind. Trends in Cognitive Sciences, 19(2), 65-72.<br><br>Schurz, M., Radua, J., Aichhorn, M., Richlan, F., Perner, J. (2014). Fractionating theory of mind: A meta-analysis of functional brain imaging studies. Neuroscience & Biobehavioral Reviews, 42, 9-34.	833600
Social imagination	Mentally representing, simulating, visualizing and reconfiguring social relationships, systems, norms, and collective futures.	You find yourself in a compromising position at work and imagine what advice your mom would give you about how to make it right	Celarent, B. & Abbott, A. (2017). Varieties of Social Imagination. Chicago: University of Chicago Press.<br><br>Hart-Brinson, P. (2016). The social imagination of homosexuality and the rise of same-sex marriage in the united states. Socius: Sociological Research for a Dynamic World, 2, 237802311663055.<br><br>Lysaker, J. T., & Miller, A. (2013). Engaging social imagination: The developmental work of wordless book reading. Journal of Early Childhood Literacy, 13(2), 147-174.<br><br>Vertovec, S. (2012). Diversity and the Social Imaginary. European Journal of Sociology, 53(3), 287-312.	39300
Imagined interactions	The process of thinking about and mentally rehearsing possible social encounters. This can include verbal exchange as well as any physical interaction or body language	A theatre audience member watching two characters negotiate a conflict on stage imaginatively tracks and anticipates how their exchange will unfold, engaging in a form of imagined interaction as an observer inhabiting the scene rather than as a participant rehearsing an encounter of their own	Friedlander, K. J. (2024). The Psychology of Creative Performance and Expertise. Routledge.<br><br>Honeycutt, J. M. (2019). Imagined Interactions and Inner Speech. Imagination, Cognition and Personality, 027623661986916.<br><br>Honeycutt, J. M., & Hatcher, L. C. (2015). Imagined Interactions. The International Encyclopedia of Interpersonal Communication, 1–10.<br><br>Vezzali, L., Capozza, D., Giovannini, D., & Stathi, S. (2011). Improving implicit and explicit intergroup attitudes using imagined contact: An experimental intervention with elementary school children. Group Processes & Intergroup Relations, 15(2), 203–212.<br><br>Honeycutt, J. M. (1990). Imagined Interactions, Imagery, and Mindfulness/Mindlessness. Mental Imagery, 121–128.	3660
Emotional imagination	To mentally simulate an emotion or event that brings about emotion	A reader absorbed in a novel may feel genuine sorrow at a character's loss or relief at their narrow escape, demonstrating that imagination can generate authentic emotional responses to fictional circumstances, not only to one's own anticipated experiences.	Friedlander, K. J. (2024). The Psychology of Creative Performance and Expertise. Routledge.<br><br>Loev, S., Inchingolo, M., & Velasco, P. F. (2022). What is it (like) to imagine an emotion?. Humanities and Social Sciences Communications, 9(1), 1-9.<br><br>Siclari, F., Baird, B., Perogamvros, L., Bernardi, G., LaRocque, J. J., Riedner, B.,... & Tononi, G. (2017). The neural correlates of dreaming. Nature neuroscience, 20(6), 872-878.<br><br>Hunter, M. (2013). Imagination may be more important than knowledge: The eight types of imagination we use. Review of Contemporary Philosophy, (12), 113-120.<br><br>Moran, R. (1994). The Expression of Feeling in Imagination. The Philosophical Review, 103(1), 75–106.	1450

Dimension	Working Definition	Example	Representative Citations	Google Scholar "Hits"
Co-imagination	Imagining shared among a group. This is commonly encountered in massively multiplayer online role playing games (MMORPG) and table top role playing games such as Dungeons and Dragons	Friends sit around a table playing Dungeons and Dragons, sharing in an imaginary world they created together where their characters are on an exhilarating campaign	Aula, I., & Masoodian, M. (2024). Creative Co-Imagination in Transgenerational Comics Workshops. Sociological Research Online, 13607804241259733.<br><br>Bowman, S. L. (2018). Immersion and shared imagination in role-playing games. In Role-playing game studies (pp. 379-394). Routledge.<br><br>Tan, E. S. H., & Visch, V. (2018). Co-imagination of fictional worlds in film viewing. Review of General Psychology, 22(2), 230-244.	247
**Sensory/Motor - Social/Emotional**				
Metaphorical imagination	The capacity to structure abstract concepts by mapping patterns of bodily experience onto them through metaphor, translating embodied knowledge into linguistic and conceptual meaning	You are working on a complicated dance choreography with your dance teacher. There is one leap you can't seem to get right. She tells you that your legs form a tightrope and you are bird sailing through the sky. After putting these words to action through your body, you are able to nail the leap every time	Stephens, V., Matthews, L., Cornelissen, J. P., & Rowlands, H. (2022). Building novel supply chain theory using “metaphorical imagination”. Journal of Supply Chain Management, 58(1), 124-139.<br><br>Gibbs, R. W. (2005). Embodiment in metaphorical imagination. Grounding cognition: The role of perception and action in memory, language, and thinking, 65, 92.	4180
Imagined speech	To simulate speaking without use of any physical movements	While preparing for your final project presentation, you envision delivering the speech in front of the class without actually moving your jaw, lips, throat, or tongue	Proix, T., Delgado Saa, J., Christen, A. et al. (2022). Imagined speech can be decoded from low- and cross-frequency intracranial Eeg features. Nature Communications, 13(48).<br><br>Lee, S. H., Lee, M., Lee, S. W. (2020). Eeg Representations of Spatial and Temporal Features in Imagined Speech and Overt Speech. In: Palaiahnakote, S., Sanniti di Baja, G., Wang, L., Yan, W. (eds) Pattern Recognition. Acpr 2019. Lecture Notes in Computer Science(), vol 12047. Springer, Cham.<br><br>Tian, X., Ding, N., Teng, X., Bai, F., Poeppel, D. (2018). Imagined speech influences perceived loudness of sound. Nature Human Behaviour, 2, 225-234.<br><br>Nguyen, C. H., K Karavas, G., Artemiadis, P. (2017). Inferring imagined speech using Eeg signals: a new approach using Riemannian manifold features. Journal of Neural Engineering, 15.<br><br>Brigham, K. & Kumar, B. V. K. V. (2010). Subject identification from electroencephalogram (Eeg) signals during imagined speech, 2010 Fourth Ieee International Conference on Biometrics: Theory, Applications and Systems (Btas), 2010, pp. 1-8.	3820
Perspective-taking	Visualizing or conjecturing what it might be like to experience from another's perspective. Related to and often utilized by theory of mind, with the exception that perspective-taking can also occur visually or kinesthetically	Seeing a construction worker on top of a tall building and imagining what it would be like to be in their place	Healey, M. L., & Grossman, M. (2018). Cognitive and affective perspective-taking: evidence for shared and dissociable anatomical substrates. Frontiers in neurology, 9, 491.<br><br>Batson, C. D., Early, S., & Salvarani, G. (1997). Perspective taking: Imagining how another feels versus imaging how you would feel. Personality and social psychology bulletin, 23(7), 751-758.	19400
**Sensory-Motor**				
Visual imagery	The act of creating mental images or pictures in one's mind without physical external stimuli	You picture your kitchen exactly as it looks right now - the counters, the cabinets, the window over the sink.	Currie, G. (1995). Visual imagery as the simulation of vision. Mind & Language, 10(1-2), 25–44.<br><br>Roland, P. E., & Gulyás, B. (1994). Visual imagery and visual representation. Trends in neurosciences, 17(7), 281–297.	280000
Motor cognition/Motor imagery	A mental performance of a physical movement	Without moving any muscles in your leg or hip, you imagine yourself kicking a soccer ball in front of you	Savaki, H. E., & Raos, V. (2019). Action Perception and Motor Imagery: Mental Practice of Action. Progress in Neurobiology.<br><br>Kim, Y. K., Park E., Lee A., Im, C-H., Kim, Y-H. (2018). Changes in network connectivity during motor imagery and execution. PLoS One. 13(1). e0190715.<br><br>O'Shea, H. & Moran, A. (2017). Does Motor Simulation Theory Explain the Cognitive Mechanisms Underlying Motor Imagery? A Critical Review. Frontiers in Human Neuroscience, 17.<br><br>Pulvermüller, F., Moseley, R. L., Egorova, N., Shebani, Z., & Boulenger, V. (2014). Motor cognition–motor semantics: Action perception theory of cognition and communication. Neuropsychologia, 55, 71-84.<br><br>Hétu, S., Grégoire, M., Saimpont, A., Coll, M. P., Eugène, F., Michon, P. E., Jackson, P. L. (2013). The neural network of motor imagery: An Ale meta-analysis. Neuroscience & Biobehavioral Reviews, 37(5), 930-949.<br><br>Gallese, V., Rochat, M., Cossu, G., Sinigaglia, C. (2009). Motor cognition and its role in the phylogeny and ontogeny of action understanding. Developmental Psychology, 45(1), 103-113.<br><br>Fuentes, C. T., & Bastian, A. J. (2007). 'Motor cognition'—what is it and is the cerebellum involved?. The Cerebellum, 6(3), 232-236.<br><br>Sommerville, J. A., & Decety, J. (2006). Weaving the fabric of social interaction: Articulating developmental psychology and cognitive neuroscience in the domain of motor cognition. Psychonomic Bulletin & Review, 13(2), 179-200.<br><br>Lotze, M. & Halsband, U.(2006). Motor imagery. Journal of Physiology-Paris, 99(4-6), 386-395.<br><br>Jackson, P. L., & Decety, J. (2004). Motor cognition: A new paradigm to study self–other interactions. Current opinion in Neurobiology, 14(2), 259-263.	202000
Musical imagination	The ability to simulate the experience of music (both novel and existing) without the presence of physical music	After going to a concert, when you go to bed and close your eyes, you still hear the melodies in your head playing repeatedly.	Martin, S., Mikutta, C., Leonard, M. K., Hungate, D., Koelsch, S., Shamma, S., Chang, E. F., del R Millán, J., Knight, R. T., Pasley, B. N. (2018). Neural Encoding of Auditory Features during Music Perception and Imagery. Cerebral Cortex, 28(12), 4222–4233.<br><br>Lima, C. F., Krishnan, C., Scott, S. K. (2016). Roles of Supplementary Motor Areas in Auditory Processing and Auditory Imagery. Trends in Neurosciences, 39(8), 527-542.<br><br>Zatorre, R. J. & Halpern, A. R. (2005). Mental Concerts: Musical Imagery and Auditory Cortex. Neuron, 47(1), 9-12.<br><br>Brodsky, W., Henik, A., Rubinstein, B. S. et al. (2003). Auditory imagery from musical notation in expert musicians. Perception & Psychophysics 65, 602–612.	7780

Dimension	Working Definition	Example	Representative Citations	Google Scholar "Hits"
Kinesthetic imagery	Without physically moving, engaging in the sensory experience of performing an action, which can also involve force, effort (regarding muscle movement, balance, and coordination), and spatial sensations	You are a sprinter and you imagine yourself in your starting position, waiting for the whistle. When the imagined race begins, you feel your heart rate increase and your fingers push off the granite track as you fly into action	Christensen, J., & Borhani, K. (2020). Dance and the Imagination: Be a Butterfly! In A. Abraham (Ed.), The Cambridge Handbook of the Imagination (Cambridge Handbooks in Psychology, pp. 620-634). Cambridge: Cambridge University Press.<br><br>Di Corrado D, Guarnera M, Vitali F, Quartiroli A, Coco M. (2019). Imagery ability of elite level athletes from individual vs. team and contact vs. no-contact sports. PeerJ.<br><br>Lotze, M. (2013). Kinesthetic imagery of musical performance. Frontiers in human neuroscience, 7, 280.<br><br>Guillot, A., Collet, C., Nguyen, V. A., Malouin, F., Richards, C., & Doyon, J. (2009). Brain activity during visual versus kinesthetic imagery: an fMRI study. Human brain mapping, 30(7), 2157-2172.<br><br>Timothy Raphael, & Joseph Roach. (2009). Activating the Kinesthetic Imagination. Transformations: The Journal of Inclusive Scholarship and Pedagogy, 20(1), 119–130.<br><br>Callow, N., & Waters, A. (2005). The effect of kinesthetic imagery on the sport confidence of flat-race horse jockeys. Psychology of Sport and Exercise, 6(4), 443-459.<br><br>Féry, Y. A. (2003). Differentiating visual and kinesthetic imagery in mental practice. Canadian Journal of Experimental Psychology/Revue canadienne de psychologie expérimentale, 57(1), 1.<br><br>Hall, C., Pongrac, J., & Buckholz, E. (1985). The measurement of imagery ability. Human Movement Science, 4(2), 107–118.	5440
Dynamic imagery	Mentally envisioning and perceiving of an action without physically engaging in output	As a basketball player you imagine your next steps to get around your opponent during the game	MacIntyre, T. E., Moran, A. P., Collet, C., & Guillot, A. (2013). An emerging paradigm: a strength-based approach to exploring mental imagery. Frontiers in Human Neuroscience.<br><br>MacIntyre, T., and Moran, A. (2010). "Meta-imagery processes among elite sports performers," in The Neurophysiological Foundations of Mental and Motor Imagery, eds A. Guillot and C. Collet (New York, Ny: Oxford University Press), 227–244.	3760
Auditory imagination	To simulate "hearing" a sound without the aid of a physical stimulus. It can be voluntary (call to mind your favorite song and play it in your head) or involuntary (when a song plays over and over in your head without your conscious prompting)	When you are feeling stressed, you imagine the rhythmic sounds of waves crashing and receding back into the vast ocean	Martin, S., Mikutta, C., Leonard, M. K., Hungate, D., Koelsch, S., Shamma, S., Chang, E. F., del R Millán, J., Knight, R. T., Pasley, B. N. (2018). Neural Encoding of Auditory Features during Music Perception and Imagery. Cerebral Cortex, 28(12), 4222–4233.<br><br>Lima, C. F., Krishnan, C., Scott, S. K. (2016). Roles of Supplementary Motor Areas in Auditory Processing and Auditory Imagery. Trends in Neurosciences, 39(8), 527-542.<br><br>Zatorre, R. J. & Halpern, A. R. (2005). Mental Concerts: Musical Imagery and Auditory Cortex. Neuron, 47(1), 9-12.<br><br>Brodsky, W., Henik, A., Rubinstein, B. S. et al. (2003). Auditory imagery from musical notation in expert musicians. Perception & Psychophysics 65, 602–612.	3000
Olfactory imagination	Imagining a particular odor, or what a particular item/object might smell like	When a coworker mentions cookies in a meeting, you imagine the smell of freshly baked chocolate chip cookies	Flohr, E. L. R., Arshamian, A., Wieser, M. J., Hummel, C., Larsson, M., Mühlberger, A., & Hummel, T. (2014). The fate of the inner nose: odor imagery in patients with olfactory loss. Neuroscience, 268, 118-127.<br><br>Krishna, A., Morrin, M., & Sayin, E. (2014). Smellizing cookies and salivating: A focus on olfactory imagery. Journal of Consumer Research, 41(1), 18-34.<br><br>Bensafi, M., Tillmann, B., Poncelet, J., Przybylski, L., & Rouby, C. (2013). Olfactory and gustatory mental imagery: modulation by sensory experience and comparison to auditory mental imagery. Multisensory imagery, 77-91.<br><br>Djordjevic, J., Zatorre, R. J., Petrides, M., Boyle, J. A., & Jones-Gotman, M. (2005). Functional neuroimaging of odor imagery. Neuroimage, 24(3), 791-801.	813
Taste imagination	The mental simulation or imagining of tastes or flavors	Imagine yourself taking a bite of a fresh lemon, you can feel the tangy citrus flavor on your tongue as if you are actually tasting it	Xiong, S., Hu, N., & Ma, Y. (2023). The influence of implied motion on expected taste perception of advertised foods: The mental simulation perspective. Food Quality and Preference, 111, 104968.<br><br>Spence, C. (2022). 'Tasting imagination': What role chemosensory mental imagery in multisensory flavour perception?. Multisensory Research, 36(1), 93-109.<br><br>Bensafi, M., Tillmann, B., Poncelet, J., Przybylski, L., & Rouby, C. (2013). Olfactory and gustatory mental imagery: modulation by sensory experience and comparison to auditory mental imagery. Multisensory imagery, 77-91.	775
Touch imagination	Imagining or simulating tactile sensations, including the feeling of touching different textures, surfaces, or objects, without actual physical contact	Imagine yourself holding silk in your left hand and sandpaper in your right. Without actually touching these materials, you can simulate the feeling of touching the two differing textures	Ruusunen, N., Hallikainen, H., & Laukkanen, T. (2023). Does imagination compensate for the need for touch in 360-virtual shopping?. International Journal of Information Management, 70, 102622.<br><br>Noheden, K. (2013). The imagination of touch: surrealist tactility in the films of Jan Švankmajer. Journal of Aesthetics & Culture, 5(1), 21111.	194
**Sensory/Motor - Cognitive/Narrative**				
Visual imagination	Using visual imagery, this involves creating visual mental representations, but is a broader constructive capacity or activity that uses such representations to create, interpret, transform, or recombine possibilities	You picture a treehouse kitchen with a stove built into a hollowed-out trunk, cabinets woven from branches, and a window looking out onto a floating garden	Wang, Y., Li, J., Wang, Z., Liang, B., Jiao, B., Zhang, P., … & Liu, M. (2021). Spontaneous activity in primary visual cortex relates to visual creativity. Frontiers in Human Neuroscience, 15.<br><br>Jankowska, D. M., Gajda, A., & Karwowski, M. (2019). How children's creative visual imagination and creative thinking relate to their representation of space. International Journal of Science Education, 41 (8), 1096-1117.<br><br>Jankowska, D. M., & Karwowski, M. (2015). Measuring creative imagery abilities. Frontiers in psychology, 6, 1591.<br><br>Atwood, G. (1971). An experimental study of visual imagination and memory. Cognitive Psychology, 2(3), 290-299.<br><br>Rimm, D. C., & Bottrell, J. (1969). Four measures of visual imagination. Behaviour research and therapy, 7(1), 63-69.	20000

Dimension	Working Definition	Example	Representative Citations	Google Scholar "Hits"
Mental rotation	The visuospatial ability to rotate a mental image around a chosen axis in 3D space	You work with a moving company and have to get a variety of furniture pieces through a small door. You have a couch that seems particularly difficult, and you mentally rotate it in the air to see which orientation will most easily get it through the door	Città, G., Gentile, M., Allegra, M., Arrigo, M., Conti, D., Ottaviano, S., & Sciortino, M. (2019). The effects of mental rotation on computational thinking. Computers & Education, 141, 103613.<br><br>Zacks, J. M. (2008). Neuroimaging studies of mental rotation: a meta-analysis and review. Journal of cognitive neuroscience, 20(1), 1-19.<br><br>Parsons, T. D., Larson, P., Kratz, K., Thiebaux, M., Bluestein, B., Buckwalter, J. G., & Rizzo, A. A. (2004). Sex differences in mental rotation and spatial rotation in a virtual environment. Neuropsychologia, 42(4), 555-562.<br><br>Marmor, G. S., & Zaback, L. A. (1976). Mental rotation by the blind: Does mental rotation depend on visual imagery? Journal of Experimental Psychology: Human Perception and Performance, 2(4), 515–521.<br><br>Shepard, R. N., & Metzler, J. (1971). Mental rotation of three-dimensional objects. Science, 171(3972), 701-703.	91500
Spatial imagery/spatial consciousness	The ability to mentally build, explore, and manipulate representations of space and form — imagining layouts, movement, or arrangements without needing to see them directly. It lets the mind "see" and reason within imagined environments, simulating scenarios, perspectives, and possibilities that aren't physically present. Includes subsets like spatial navigation and scene construction.	As you imagine your bedroom, you have a sense of how far your bed is from the desk and where everything in the room lies in relation to it. You are able to construct the scene of the bedroom, and how you or others might navigate through it.	Galland, D. and Grønning, M. (2019). Spatial consciousness. The Wiley Blackwell Encyclopedia of Urban and Regional Studies, 1-9.<br><br>El Haj, M., & Lenoble, Q. (2018). Eying the future: Eye movement in past and future thinking. Cortex, 105, 97–103.<br><br>Campos, A. (2013). Reliability and Percentiles of a Measure of Spatial Imagery. Imagination, Cognition and Personality, 32(4), 427–431.<br><br>Thompson, W. L., Slotnick, S. D., Burrage, M. S., & Kosslyn, S. M. (2009). Two forms of spatial imagery: Neuroimaging evidence. Psychological science, 20(10), 1245-1253.<br><br>Vannucci, M., & Mazzoni, G. (2009). Individual differences in object and spatial imagery: Personality correlates. Personality and Individual Differences, 46(4), 402–405.<br><br>Buckner, R. L., & Carroll, D. C. (2007). Self-projection and the brain. Trends in cognitive sciences, 11(2), 49-57.<br><br>Hassabis, D., & Maguire, E. A. (2007). Deconstructing episodic memory with construction. Trends in cognitive sciences, 11(7), 299-306.	17200
Verbal imagery/Inner speech	Imagining producing speech or words without vocalizing it; silently articulating a word or sentence	You imagine your family and friends verbally congratulating you on getting into your dream school	Fernyhough, C., & Borghi, A. M. (2023). Inner speech as language process and cognitive tool. Trends in cognitive sciences, 27(12), 1180-1193.<br><br>Alderson-Day, B., & Fernyhough, C. (2015). Inner speech: Development, cognitive functions, phenomenology, and neurobiology. Psychological bulletin, 141(5), 931.<br><br>Morin, A. (2005). Possible links between self-awareness and inner speech theoretical background, underlying mechanisms, and empirical evidence. Journal of Consciousness Studies, 12(4-5), 115-134.<br><br>Shergill, S., Bullmore, E., Brammer, M., Williams, S., Murray, R., & McGuire, P. (2001). A functional study of auditory verbal imagery. Psychological Medicine, 31(2), 241-253.<br><br>McGuire, P., Silbersweig, D., Murray, R., David, A., Frackowiak, R., & Frith, C. (1996). Functional anatomy of inner speech and auditory verbal imagery. Psychological Medicine, 26(1), 29-38.	17700
Embodied imagination	Feeling, in the body, what you are imagining	You are visiting your friend in the hospital who just broke their foot and femur in a skateboarding accident. Looking at them, you cringe while imagining how it must have felt falling off their board onto the road and the edge of the curb and you feel your own foot and leg emit a pang of pain	Clavel Vázquez, M. J., & Clavel-Vázquez, A. (2023). Robustly embodied imagination and the limits of perspective-taking. Philosophical Studies, 180(4), 1395-1420.<br><br>Langland-Hassan, P. (2020). Explaining imagination. Oxford University Press.	3340
Linguistic imagination	The capacity to use language generatively, constructing, extending, and transforming meaning beyond the literal through word choice, syntax, narrative, and figuration. It is the ability to deploy language not merely to describe or communicate, but to bring into being possible worlds, perspectives, and meanings that would not otherwise exist	You are a poet and attempt to construct new language combinations and variations in rhythm to create an emotionally heartfelt poem	Kearney, R. (2018). Exploring Imagination. Stretching the Limits of Productive Imagination: Studies in Kantianism, Phenomenology and Hermeneutics, 187.<br><br>Dor, D. (2015). The instruction of imagination: Language as a social communication technology. Oxford University Press.<br><br>Miller, J. E. (1970). The linguistic imagination. Elementary English, 47(4), 467-475.	1590
Internal visual imagery	Visual imagery from the first-person perspective; viewing the imagined scenario as if through your own eyes	Imagine you are a lioness, seeing from her perspective, honing in on her prey and slow stalking towards it	Yu, Q. H., Fu, A. S., Kho, A., Li, J., Sun, X. H., & Chan, C. C. (2016). Imagery perspective among young athletes: Differentiation between external and internal visual imagery. Journal of Sport and Health Science, 5(2), 211-218.<br><br>Jiang, D., Edwards, M. G., Mullins, P., & Callow, N. (2015). The neural substrates for the different modalities of movement imagery. Brain and Cognition, 97, 22-31.<br><br>Callow, N., Roberts, R., Hardy, L., Jiang, D., & Edwards, M. G. (2013). Performance improvements from imagery: evidence that internal visual imagery is superior to external visual imagery for slalom performance. Frontiers in human neuroscience, 7, 697.<br><br>Hardy, L., & Callow, N. (1999). Efficacy of external and internal visual imagery perspectives for the enhancement of performance on tasks in which form is important. Journal of Sport and Exercise Psychology, 21(2), 95-112.<br><br>White, A., & Hardy, L. (1995). Use of different imagery perspectives on the learning and performance of different motor skills. British Journal of Psychology, 86(2), 169-180.	1100

Dimension	Working Definition	Example	Representative Citations	Google Scholar "Hits"
External visual imagery	Ability to imagine yourself from a third-person perspective, seeing yourself as the focus of your imagination (rather than a first-person perspective)	While planning your outfit for a big presentation, you imagine what the outfit will look like on you and visualize yourself from a third-person perspective	Montuori, S., Curcio, G., Sorrentino, P., Belloni, L., Sorrentino, G., Foti, F., & Mandolesi, L. (2018). Functional role of internal and external visual imagery: preliminary evidences from pilates. Neural plasticity, 2018.<br><br>Yu, Q. H., Fu, A. S., Kho, A., Li, J., Sun, X. H., & Chan, C. C. (2016). Imagery perspective among young athletes: Differentiation between external and internal visual imagery. Journal of Sport and Health Science, 5(2), 211-218.<br><br>Callow, N., & Hardy, L. (2004). The relationship between the use of kinaesthetic imagery and different visual imagery perspectives. Journal of sports sciences, 22(2), 167-177.<br><br>Hardy, L., & Callow, N. (1999). Efficacy of external and internal visual imagery perspectives for the enhancement of performance on tasks in which form is important. Journal of Sport and Exercise Psychology, 21(2), 95-112.	732
Experiential imagination	The generation of a mental experience that includes many sensory modalities that mimic being physically present in the mental scene; seeing, hearing, feeling, smelling, etc. parts of the scene/experience	You imagine what it would be like to ride on the back of a dragon flying over a mountain range; what you would experience through your senses, what emotions you would feel, and how you might behave	Clavel Vázquez, M. J., & Clavel-Vázquez, A. (2023). Robustly embodied imagination and the limits of perspective-taking. Philosophical Studies, 180(4), 1395-1420.<br><br>Liefke, K., & Werning, M. (2020, November). Experiential imagination and the inside/outside-distinction. In Jsai International Symposium on Artificial Intelligence (pp. 96-112).<br><br>Kind, A. (Ed.). (2016). The Routledge handbook of philosophy of imagination (Vol. 12). London: Routledge.<br><br>Dokic, J., & Arcangeli, M. (2014). The heterogeneity of experiential imagination. In Open Mind. Open Mind. Frankfurt am Main: Mind Group Springer, Cham.	265
Creative visual imagination	The process of visualizing, transforming, attenuating old mental representations and creating a new one.	An architect staring at a blank site sketches several unconventional building forms, mentally rotating and combining shapes (a spiral staircase merged with a cantilevered roofline, say) that don't exist yet, then evaluates which combinations are structurally feasible and aesthetically novel before committing to a design.	Wang, Y., Li, J., Wang, Z., Liang, B., Jiao, B., Zhang, P., … & Liu, M. (2021). Spontaneous activity in primary visual cortex relates to visual creativity. Frontiers in Human Neuroscience, 15.<br><br>Jankowska, D. M., & Karwowski, M. (2015). Measuring creative imagery abilities. Frontiers in psychology, 6, 1591.	146
Interoceptive imagination	The capacity to mentally simulate, anticipate, or re-create internal bodily sensations, such as breath, heartbeat, tension, warmth, nausea, calm, or hunger, in the absence of the actual physiological stimulus	Imagining the feeling of nausea in response to food poisoning or a noxious substance	Parra, A., & Rey, A. (2019). The interoception and imagination loop in hypnotic phenomena. Consciousness and Cognition, 73, 102765.<br><br>Wilson-Mendenhall, C. D., Henriques, A., Barsalou, L. W., & Barrett, L. F. (2019). Primary interoceptive cortex activity during simulated experiences of the body. Journal of cognitive neuroscience, 31(2), 221-235.	5
**CATEGORIES**				
	**Cross-Domain**			
	**Cognitive-Narrative**			
	**Cognitive/Narrative -Social/Emotional**			
	**Social-Emotional**			
	**Sensory/Motor - Social/Emotional**			
	**Sensory-Motor**			
	**Sensory/Motor - Cognitive/Narrative**			
