# Supplementary material for: Defining imagination: a conceptual mapping and provisional model
Source: Front Psychol. 2026 Sep 21;17:1888826. doi: 10.3389/fpsyg.2026.1888826 (PMC13639878; doi:10.3389/fpsyg.2026.1888826)
Supplement: Supplementary file 3 [file Data_Sheet_3.pdf]

Applied and Disciplinary Forms of Imagination	Working Definition	Example	Representative Citations	Google Scholar "Hits"
These forms of imagination name a professional, commercial, or disciplinary context of application rather than a distinct cognitive mechanism, phenomenological modality, or content type. They are presented separately from the core taxonomy (Supplementary Materials A and B) for this reason. This list is illustrative rather than exhaustive: other disciplinary labels (e.g., historical, legal, architectural, political, cinematic, or literary imagination) would likely qualify on the same grounds had they appeared with comparable frequency in the literature search.				
Sociological imagination	To understand how social systems can influence the individual. It involves looking beyond personal experiences to see how larger societal factors influence problems and offer opportunities for change	Consider yourself being a recent college graduate who's struggling to find a job. Instead of blaming yourself for the unemployment, you analyze the market and observe social factors that result in an increase in unemployment rate, which could explain this broader trend	Mills C. W. (2000). The sociological imagination. Oxford University Press<br><br>William Ayers (1995) Social imagination: a conversation with Maxine Greene, International Journal of Qualitative Studies in Education, 8:4, 319-328	78500
Geographical imagination	The awareness and perception of objects and environments in space in relation to oneself, including the ability to navigate the world around us	You are meeting your friend downtown and finally find parking away from your rendezvous point. Calling up your buddy, you let them know that, judging from the tall building you can see in the distance where you planned to meet, you are about 10 minutes away and heading there on foot right now	Gieseking, J. J. (2017). The Geographical Imagination. In International Encyclopedia of Geography (eds. D. Richardson, N. Castree, M. Goodchild, A. Jaffrey, W. Liu, A. Kobayashi, and R. Marston). New York: Wiley-Blackwell and the Association of American Geographers<br><br>Daniels, S. (2011). Geographical imagination. Transactions of the Institute of British Geographers, 36(2), 182-187<br><br>Gieseking, J. (2007). Understanding the Geographical Imagination. The Website of Jack Jen Gieseking	18200
Scientific imagination	Mentally creating new ideas and concepts that are aligned with scientific rules and principles and applicable to daily life	You are an engineer imagining a new design for airplane seats for international flights that will be comfortable for sitting and sleeping	Nur Zuraimi Zam Jamar and Siew, Nyet Moi (2020) Investigating the Validity and Reliability of a Scientific Imagination Test for Tenth graders. International Journal of Educational Technology and Learning, 8(2), 52-60. Issn 2523-0581<br><br>Wang., C.-C., Ho, H.-C., Wu, J.-J., & Cheng, Y.-Y. (2014). Development of the scientific imagination model: A concept-mapping perspective. Thinking Skills and Creativity, 13, 106-119	17200
Mathematic imagination	The ability to visualize and manipulate abstract mathematical concepts	You are asked to solve a dice probability problem where you have to calculate the probability of rolling a 1. To do this, you represent a six-sided dice in your imagination and see all sides have equal probability, you then get 1/6 as your answer	Handelman, Matthew, The Mathematical Imagination: On the Origins and Promise of Critical Theory (New York, Ny, 2019; online edn, Fordham Scholarship Online, 23 Jan. 2020), accessed 4 May 2023<br><br>Irakleous, P., Christou, C. & Pitta-Pantazi, D. Mathematical imagination, knowledge and mindset. Zdm Mathematics Education 54, 97-111 (2022)<br><br>Nemirovsky, R., & Ferrara, F. (2009). Mathematical imagination and embodied cognition. Educational Studies in Mathematics, 70, 159-174<br><br>Lakoff, G., & Nunez, R. (2000). Where Mathematics Comes From: How the Embodied Mind Brings Mathematics into Being. Basic Books	2570

Applied and Disciplinary Forms of Imagination	Working Definition	Example	Representative Citations	Google Scholar "Hits"
These forms of imagination name a professional, commercial, or disciplinary context of application rather than a distinct cognitive mechanism, phenomenological modality, or content type. They are presented separately from the core taxonomy (Supplementary Materials A and B) for this reason. This list is illustrative rather than exhaustive: other disciplinary labels (e.g., historical, legal, architectural, political, cinematic, or literary imagination) would likely qualify on the same grounds had they appeared with comparable frequency in the literature search.				
Entrepreneurial imagination	The ability to construct a future undertaking by identifying where a service or goods may be lacking and creating innovative solutions to build resources for those goods/services	After a recurring hand cramp while working at the computer, you imagine a novel ergonomic mouse design	Gartner, W. B., Nordqvist, M., Suddaby, R., & Schultz, J. L. (2025). Fiction and the entrepreneurial imagination. Entrepreneurship & Regional Development, 1-15<br><br>Chiles, T. H., Crawford, B., & Elias, S. R. (2021). Mind, body, and soul: A spiritual perspective on the entrepreneurial imagination. Organization Theory, 2(2), 26317877211005786	1690
Strategic imagination	Using visualization, simulation, or other forms of imagination to identify and evaluate opportunities and their pros and cons, in order to make an optimal decision about a future event	You are a marketing analyst at a company looking to expand into a new market. You explore ways to take your place in comparison to key existing players and maximize returns by imagining all market competitors as players on a football field	Hunter, M. (2013). Imagination may be more important than knowledge: The eight types of imagination we use. Review of Contemporary Philosophy, (12), 113-120	1590
Consumer imagination	Imagining what it would be like to use a product you have not physically interacted with	When watching an ad about a face cream, you imagine what it would be like to have that face cream and how glowy your skin will be after using it	Dewi, I. J., & Ang, S. H. (2015). The conceptualization and development of advertisement-evoked imagination scale. Asia Marketing Journal, 17 (2), 2<br><br>Hung, I. W., & Wyer, R. S., Jr. (2011). Shaping consumer imaginations: The role of self-focused attention in product evaluations. Journal of Marketing Research, 48(2), 381-392<br><br>Molesworth, M., & Denegri-Knott, J. (2007). Digital Play and the Actualization of the Consumer Imagination. Games and Culture, 2(2), 114-133<br><br>Hope Jensen Schau (2000),"Consumer Imagination, Identity and Self-Expression", in Na - Advances in Consumer Research Volume 27, eds. Stephen J. Hoch and Robert J. Meyer, Provo, Ut : Association for Consumer Research, Pages: 50-56	1140

Applied and Disciplinary Forms of Imagination	Working Definition	Example	Representative Citations	Google Scholar "Hits"
These forms of imagination name a professional, commercial, or disciplinary context of application rather than a distinct cognitive mechanism, phenomenological modality, or content type. They are presented separately from the core taxonomy (Supplementary Materials A and B) for this reason. This list is illustrative rather than exhaustive: other disciplinary labels (e.g., historical, legal, architectural, political, cinematic, or literary imagination) would likely qualify on the same grounds had they appeared with comparable frequency in the literature search.				
Requisite imagination	Anticipating possible errors or faults of newly designed systems and coming up with solutions to account for those pitfalls	You are a part of a group of architects that are building a bridge in Louisiana. Given its usual warm weather and swampy land conditions, you collectively anticipate situations where rain and failures of sewer systems combined with the climate characteristics may cause problems to the bridge, allowing you to therefore carefully select the most appropriate material and structure of the bridge before building	Sittig, D. F., Lakhani, P., Singh, H. (2022). Applying requisite imagination to safeguard electronic health record transitions. Journal of the American Medical Informatics Association, 29(5), 1014-1018<br><br>Love, P. E. D. & Matthews, J. (2020). Quality, requisite imagination and resilience: Managing risk and uncertainty in construction. Reliability Engineering & System Safety, 204, 107172<br><br>Arnoud, J. (2019). The Contribution of Creativity of Action to Safety: The Key Role of Requisite Imagination. In: Bagnara, S., Tartaglia, R., Albolino, S., Alexander, T., Fujita, Y. (Eds.), Proceedings of the 20th Congress of the International Ergonomics Association (Iea 2018). Advances in Intelligent Systems and Computing, vol 821. Springer, Cham<br><br>Westrum, R. (1993). Cultures with requisite imagination. In Verification and Validation of Complex Systems: Human Factors Issues (pp. 401-416). Springer Berlin Heidelberg	954
